# Elucidating the distinct contributions of miR-122 in the HCV life cycle reveals insights into virion assembly

**DOI:** 10.1093/nar/gkad094

**Published:** 2023-02-21

**Authors:** Marylin Rheault, Sophie E Cousineau, Danielle R Fox, Quinn H Abram, Selena M Sagan

**Affiliations:** Department of Microbiology & Immunology, McGill University, Montréal, Canada; Department of Microbiology & Immunology, McGill University, Montréal, Canada; Department of Microbiology & Immunology, McGill University, Montréal, Canada; Department of Physiology, McGill University, Montréal, Canada; Department of Biochemistry, McGill University, Montréal, Canada; Department of Microbiology & Immunology, McGill University, Montréal, Canada; Department of Biochemistry, McGill University, Montréal, Canada

## Abstract

Efficient hepatitis C virus (HCV) RNA accumulation is dependent upon interactions with the human liver-specific microRNA, miR-122. MiR-122 has at least three roles in the HCV life cycle: it acts as an RNA chaperone, or ‘riboswitch’, allowing formation of the viral internal ribosomal entry site; it provides genome stability; and promotes viral translation. However, the relative contribution of each role in HCV RNA accumulation remains unclear. Herein, we used point mutations, mutant miRNAs, and HCV luciferase reporter RNAs to isolate each of the roles and evaluate their contribution to the overall impact of miR-122 in the HCV life cycle. Our results suggest that the riboswitch has a minimal contribution in isolation, while genome stability and translational promotion have similar contributions in the establishment phase of infection. However, in the maintenance phase, translational promotion becomes the dominant role. Additionally, we found that an alternative conformation of the 5′ untranslated region, termed SLII^alt^, is important for efficient virion assembly. Taken together, we have clarified the overall importance of each of the established roles of miR-122 in the HCV life cycle and provided insight into the regulation of the balance between viral RNAs in the translating/replicating pool and those engaged in virion assembly.

## Graphical Abstract

**Graphical Abstract:**
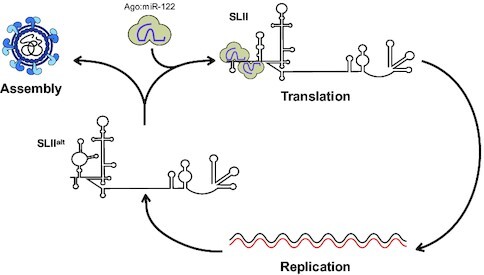
The conformation of the HCV 5' UTR and interactions with Ago:miR-122 complexes control the fate of newly-synthesized viral RNAs. The energetically favorable SLII^alt^ conformation can engage in viral packaging, while spontaneous or Ago:miR-122-mediated conversion to the SLII conformation, commits the viral RNA to translation.

## INTRODUCTION

Hepatitis C virus (HCV) affects approximately 71 million people worldwide and typically results in a chronic infection that can lead to steatosis, cirrhosis and hepatocellular carcinoma (1–3). The ∼9.6 kb positive-sense HCV genomic RNA encodes a single open-reading frame flanked by highly structured 5′ and 3′ untranslated regions (UTRs) that contain several *cis*-acting RNA elements with important roles in the viral life cycle (4). Specifically, the 5′ UTR contains the internal ribosomal entry site (IRES), composed of stem-loops (SL) II-IV, which directs cap-independent translation of the viral polyprotein (5). Additionally, SLI-II in the 5′ UTR as well as the 3′ UTR (including the variable region, polyU/UC-tract and 98-nt X-tail) are required for viral RNA replication (6–10). The 5′ UTR of the HCV genome also contains two conserved binding sites for the human liver-specific microRNA (miRNA), miR-122 (11–13).

**Figure 1.**
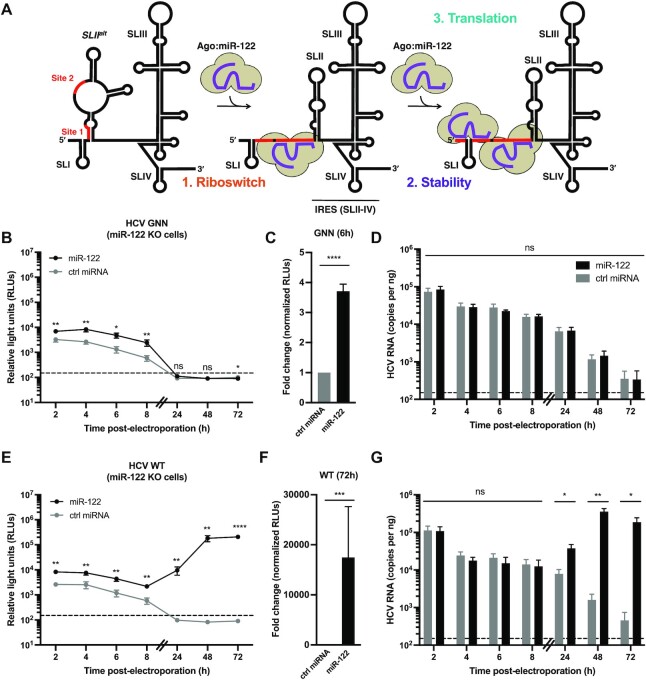
Overall contributions of miR-122’s riboswitch, genome stability and translational promotion activities. (**A**) Schematic representation of the three roles attributed to miR-122 in the HCV life cycle. In the absence of miR-122, WT HCV RNA favors the SLII^alt^ conformation, and recruitment of miR-122 to site 2 promotes RNA chaperone or riboswitch activity, allowing the viral internal ribosomal entry site (IRES) to form (including SLII-IV). Subsequent recruitment of a second miR-122 molecule to site 1 provides genome stability (protecting the 5′ triphosphate from pyrophosphatases and exoribonuclease-mediated decay), and translational promotion [through interactions between the Argonaute (Ago) protein at site 2 and the viral IRES]. (B–G) Full-length RLuc HCV RNAs were co-electroporated into miR-122 knockout (KO) cells with miR-122 or control (ctrl) miRNA, and a capped Firefly luciferase (FLuc) reporter RNA. Half the number of cells were plated for the 24–72 h time points and the 48–72 h lysates were diluted 2-fold to ensure values were within the range of the luciferase assay. RLuc activities for (**B**, **C**) GNN and (**E**, **F**) WT viral RNAs were monitored over time. The limit of detection is indicated. Data in (B) and (E) are representative data from one of five independent biological replicates with three technical replicates, and error bars represent the standard error of the mean (SEM). In (C) and (F), the RLuc activity for GNN or WT HCV RNAs across all independent biological replicates at 6 or 72 h, respectively, normalized to the FLuc (transfection efficiency) control at 2 h, were used to calculate the fold change, with the control miRNA condition set to 1. Data is displayed as the mean of four (GNN) or six (WT) independent biological replicates, and error bars represent the SEM. Viral RNA levels for (**D**) GNN and (**G**) WT HCV were monitored by RT-qPCR. HCV RNA copy numbers were quantified using a standard curve and normalized to the GAPDH internal control. The limit of detection is indicated. Data is displayed as the mean of four independent biological replicates (except for the 48h WT + miR-122, 72h WT + ctrl miRNA and 4h GNN + ctrl miRNA which represent three independent biological replicates), with error bars corresponding to the SEM. Statistical significance was determined by multiple Student's *t* test, ****P ≤ 0.0001; *** *P* ≤ 0.001; **P ≤ 0.01; *P ≤ 0.05 ns, not significant (*P* ≥ 0.05).

miR-122 is highly conserved across the vertebrate lineage and specifically expressed in the liver, with ∼135 000 copies per hepatocyte (12,14). While the canonical function of miR-122 is in regulation of cholesterol synthesis and fatty acid metabolism, miR-122 interacts with two sites (site 1 and site 2) at the 5′ terminus of the HCV genome, and these interactions promote viral RNA accumulation (11–13,15). Several recent studies have revealed a new model for miR-122:HCV RNA interactions that suggests that miR-122 has at least three roles in the HCV life cycle (Figure 1A) (16–20). Firstly, upon entry into the cell, the viral 5′ UTR is thought to adopt the most energetically favorable conformation (termed SLII^alt^), in which only the second miR-122 binding site (site 2) is accessible. As such, an Argonaute (Ago):miR-122 complex is likely first recruited to site 2 of the HCV genome. This results in an RNA chaperone-like switch in conformation, akin to a riboswitch, converting SLII^alt^ to the SLII structure, thereby allowing the viral IRES (SLII-IV) to form (17,19,20). This conformational change then reveals site 1, allowing recruitment of a second Ago:miR-122 complex, which promotes genome stability by base pairing with the 5′ terminus, protecting the viral RNA from cellular pyrophosphatase activity and subsequent exoribonuclease-mediated decay (13,16,21–24). Finally, the Ago protein bound to site 2 makes direct contact with the IRES at SLII-III, further stabilizing the IRES and promoting viral translation (Figure 1A) (16,17,19,21–26).

Herein, we explored the importance of each of the three roles attributed to miR-122, namely the riboswitch, genome stabilization, and translational promotion activities, to the overall impact of miR-122 in the HCV life cycle. To do so, we used a combination of luciferase reporter viral RNAs with point mutations and compensatory miR-122 molecules, which allowed us to study each of the three roles in isolation. We found that the relative contributions of each of the three roles differed during the establishment and maintenance phases of the viral life cycle. Specifically, during the establishment phase of the viral life cycle (i.e. before viral replication is established), our results suggest that the riboswitch activity alone does not play a significant role, while genome stabilization and translational enhancement contribute similarly to translation and viral RNA accumulation. However, during the maintenance phase (i.e. after several rounds of viral replication), genome stabilization becomes less important and translational promotion contributes predominantly to viral RNA accumulation. Interestingly, since we observed that the riboswitch activity did not have a significant effect on viral RNA accumulation using luciferase reporter RNAs, we decided to explore the importance of the riboswitch activity using the fully packaging-competent cell culture-derived HCV (HCVcc) system. By using point mutations that destabilized the SLII^alt^ conformation and stabilized the SLII conformation, we observed a progressive decrease in infectious particle production, suggesting that SLII^alt^ is required for efficient viral packaging. Thus, our results provide a new model whereby SLII^alt^ and miR-122 interactions with the viral 5′ UTR regulate the balance of viral RNAs in the translating/replicating pool and those engaged in virion assembly.

## MATERIALS AND METHODS

### Biological resources

Parental and miR-122 knockout (KO) Huh-7.5 human hepatoma cells were provided by Drs. Charlie Rice and Matthew Evans, respectively (27,28). All cells were maintained in Dulbecco's minimal essential medium (DMEM) supplemented with 10% fetal bovine serum, 1× MEM nonessential amino acids, and 2 mM l-glutamine. All cells were maintained at 37°C/5% CO_2_ and were routinely screened for mycoplasma contamination.

The pJ6/JFH-1 full-length (FL) *Renilla* luciferase (RLuc) wild-type (WT) and GNN plasmids bear full-length viral sequences derived from the J6 (structural genes) and Japanese Fulminant Hepatitis-1 (JFH-1, nonstructural genes) genotype 2 isolates of HCV, with a RLuc reporter inserted between the p7 and NS2-coding regions (29). The pJ6/JFH-1 FL RLuc GNN plasmid also bears inactivating mutations (GDD → GNN) within the NS5B RNA polymerase active site (29). The S1:p3 (C26A), S2:p3 (C41A) and additional point mutations (U4C, G20A, and G28A) were generated by overlapping PCR and were subcloned into the J6/JFH-1 plasmid using the *EcoRI* and *KpnI* restriction sites (18). The pJFH-1_T_ plasmid encodes a cell culture-adapted JFH-1 with three adaptive mutations that increase viral titers in cell culture (30). The JFH-1_T_ U4C, G20A and G28A mutations were similarly generated by overlapping PCR and were subcloned into the JFH-1 _T_ plasmid using the *EcoRI* and *AgeI* restriction sites.

### Reagents

miR-122: 5′-UGG AGU GUG ACA AUG GUG UUU GU-3′, miR-122*: 5′-AAA CGC CAU UAU CAC ACU AAA UA-3′, miR-122p3U: 5′-UGU AGU GUG ACA AUG GUG UUU GU-3′, ctrl miRNA: 5′-UAA UCA CAG ACA AUG GUG UUU GU-3′, and ctrl miRNA*: 5′-AAA CGC CAU UAU CUG UGA GGA UA-3′ were all synthesized by Integrated DNA Technologies (13). MicroRNA duplexes were diluted to a final concentration of 20 μM in RNA annealing buffer (150 mM HEPES pH 7.4, 500 mM potassium acetate, 10 mM magnesium acetate). After a denaturation step of 1 min (min) at 95°C, miRNAs were annealed at 37°C for 1 h, and stored at –20°C or –80°C until use. The same passenger strand (miR-122*) was annealed with both miR-122 and miR-122 p3U guide strands.

### *In vitro* transcription

To make full-length viral RNAs, all templates were linearized with *XbaI* and *in vitro* transcribed using T7 RNA polymerase (NEB). Briefly, 1 μg of linearized template DNA was incubated at 30°C for 1 h 10 min with 1 mM each ATP, UTP and CTP, 1.2 mM GTP, 0.8 U/μl RiboLock RNase inhibitor (ThermoFisher Scientific) and 200 U T7 RNA polymerase in a final volume of 50 μl, followed by a 20 min DNase I (NEB) digest at 37°C. Capped Firefly luciferase (FLuc) mRNAs were generated from the pT7Luc plasmid (Promega) linearized with *XmnI* and were *in vitro* transcribed using the mMESSAGE mMACHINE T7 Transcription Kit (Invitrogen) according to the manufacturer's instructions. *In vitro* transcribed RNAs were precipitated in 0.1 volume of 3M NaOAc pH 5.2 and 2.5 volumes 95–100% ethanol with 1 μl GlycoBlue™ co-precipitant (ThermoFisher Scientific) and stored at –80°C until use.

### Electroporations

Electroporations were carried out as previously described (16,31). Briefly, for collection of luciferase data only, 4 $ \times$ 10^6^ cells resuspended in 400 μl cold phosphate-buffered saline (PBS; Wisent) were mixed with 10 μg WT or GNN J6/JFH-1 FL RLuc RNAs, 1 μg capped FLuc mRNA, and in some cases 60 pmol duplexed miR-122 molecules (wild-type, ctrl or p3U) and electroporated using 4 mm cuvettes at 270 V, 950 μF and infinite resistance, optimized for the Bio-Rad Gene Pulser XCell (Bio-Rad). Electroporated cells were resuspended in 7.5 ml media and 1 ml per time point were plated in 12-well plates for luciferase assays. For replication competent assays, only 0.5 ml was plated for the 24–72 h time points. All experiments were carried out in at least three independent biological replicates. The technical replicate(s) within the biological replicates were performed in a unique cuvette, that was electroporated with *in vitro* transcribed HCV RNA from separate reactions.

For simultaneous luciferase assays with RT-qPCR, 8 $ \times$ 10^6^ cells were used in electroporations and 3 cuvettes were pooled in 7 ml media. Subsequently, 200 μl per time point was plated in 12-well plates for luciferase assays (100 μl for 24–72 h time point in replication-competent assays), and 1 ml (2–8 h) or 0.5 ml (24–72 h) per time point were plated in 6-well plates for RT-qPCR analysis. Each experiment was performed in at least three independent biological replicates. The data was examined to verify that the trend observed in each of the independent biological replicates was the same, and one of the biological replicates, with three technical replicates performed therein, is shown as a representative plot. However, all fold-change calculations were made using data from all three (or more, where indicated) independent biological replicates.

### Luciferase assays

For luciferase assays, cells were washed in PBS and harvested in 200 μl (400 μl for 48–72 h time points in replication-competent assays) of 1× passive lysis buffer (Promega). Luciferase data was analyzed using the Dual Luciferase Assay kit (Promega) according to the manufacturer's instructions, with the modification that 25 μl of reagent was used per 10 μl of sample. Luciferase assays were performed with a 20/20 luminometer with an integration time of 10 s and each sample was read in duplicate.

### Reverse transcription quantitative polymerase chain reaction (RT-qPCR) analyses

For analysis of JFH-1_T_ WT and RAV accumulation, the iTaq Universal Probes One-Step kit (Bio-Rad) was used to perform duplex assays probing for the HCV genome with primers NS5B-FOR (5′-AGA CAC TCC CCT ATC AAT TCA TGG C-3′) and NS5B-REV (5′-GCG TCA AGC CCG TGT AAC C-3′) and NS5B-FAM probe (5′-ATG GGT TCG CAT GGT CCT AAT GAC ACA C-3′) as well as a GAPDH loading control (PrimePCR Probe assay with HEX probe, Bio-Rad) (32). Each 20 μl reaction contained 500 ng of total RNA, 1.5 μl of the HCV primers and probe, and 0.5 μl of the GAPDH primers and probe. All RT-qPCR reactions were conducted in a CFX96 Touch Deep Well Real-Time PCR system (Bio-Rad). Genome copies were calculated against a genomic RNA standard curve, and fold-differences in gene expression were calculated using the 2^–ΔΔCt^ method (33).

For strand-specific RT-qPCR analysis, electroporated Huh-7.5 or miR-122 KO cells plated in 6-well plates were lysed in 500 μl TRIzol reagent (ThermoFisher Scientific) and total RNA was extracted according to the manufacturer's instructions. Reverse transcription was performed using the HCV-specific primer: Tag4-NS5B-REV: 5′-GAA GCT GAC TTG ACA TGT TGC CGC GTC AAG CCC GTG TAA CC-3′ as well as the human GAPDH-specific primer: hsGAPDH RT: 5′- GCT CCT GGA AGA TGG TGA TGG GAT TTC C-3′ (32,34). Briefly, 500 ng total RNA was incubated with 100 nM primers and dNTP mix (50 μM each) at 95°C for 5 min to denature the sample. The mixture was then cooled to 55°C prior to addition of reverse transcriptase buffer, 40 U RiboLock (ThermoFisher Scientific) and 100 U Maxima H Minus reverse transcriptase. The reaction was incubated at 55°C for 30 min for cDNA synthesis, and heat inactivated at 85°C for 15 min. The cDNA was subsequently purified using the DNA clean and concentrator kit (Zymo) according to manufacturer's instructions, with the inclusion of the optional RNA hydrolysis step, and samples were eluted in 10 μl of RNase-free water. Subsequently, 3 μl was used for qPCR (in duplicate) using the iTaq Universal Probes Supermix (Bio-Rad) according to manufacturer's instructions using 400 nM each Tag4 (5′-GAA GCT GAC TTG ACA TGT TGC C-3′) and NS5B-FOR and 200 nM of the NS5B-FAM probe (32). GAPDH was simultaneously detected using 150 nM each hsGAPDH-FOR (5′-GGA AGG TGA AGG TCG GAG TCA ACG G-3′), hsGAPDH-REV (5′-GCT CCT GGA AGA TGG TGA TGG GAT TTC C-3′) and GAPDH-HEX probe (5′-AGC TTC CCG TTC TCA GCC TTG AC-3′) (34). Genome copies were calculated against a genomic RNA standard curve, and normalized to the GAPDH internal control using the 2^–ΔΔCt^ method (33).

### RNA structure prediction

To predict the secondary structures and Gibb's free energy (Δ*G*) of the first 117 nucleotides of the JFH-1 sequence, the RNAstructure 6.4 secondary structure prediction software was accessed from the Matthews lab server https://rna.urmc.rochester.edu/RNAstructureWeb/ (35). To avoid spurious interactions that artificially decreased the calculated Δ*G*, the first three nucleotides of the sequence were constrained to be single-stranded. The results were saved as dot bracket files and used to generate the predicted structures with the RNA2Drawer browser app https://rna2drawer.app/ (36).

### Focus-forming unit (FFU) assays

One day prior to infection, 8-well chamber slides (Lab-Tek) were seeded with 4 $ \times$ 10^5^ Huh-7.5 cells/well. Infections were performed with 10-fold serial dilutions of viral samples in 100 μl for 4 h, after which the supernatant was replaced with fresh media. Three days post-infection, slides were fixed in 100% acetone and stained with anti-HCV core antibody (1:100, clone B2, Anogen), and subsequently with the AlexaFluor-488-conjugated anti-mouse antibody (1:200, ThermoFisher Scientific) for immunofluorescence analysis. Viral titers are expressed as the number of focus-forming units (FFU) per ml. Extracellular virus titers were determined directly from cell supernatants.

### Statistical analyses

Statistical analyses were performed using GraphPad Prism v9 (GraphPad, USA). For analysis of simultaneous triplicate luciferase assays and strand-specific RT-qPCR results, multiple unpaired Student's *t* test was performed for each time point, with the assumptions of Gaussian distribution and same standard deviation for samples at the same time points. For analysis of fold changes across independent replicates, ratio paired Student's *t* test was performed for each time point.

## RESULTS

### Overall contribution of miR-122 to HCV translation and viral RNA accumulation.

To determine the overall contribution of miR-122 to HCV translation and viral RNA accumulation, we co-electroporated full-length replication-competent (WT) and replication-defective (GNN) HCV *Renilla* luciferase (RLuc) reporter RNAs and miR-122 or control miRNA duplexes into miR-122 knockout (KO) cells and monitored luciferase activity over time (Figure 1 and Supplementary Figures S1 and S2). Luciferase activity was used as a proxy for viral RNA accumulation and/or decay over time. As shown previously, introduction of HCV reporter RNAs into miR-122 KO cells in the absence of exogenously provided miR-122 resulted in transient translation of the input RNA from 2–8 h post-electroporation. However, because miR-122 is required for efficient viral RNA accumulation, the signal quickly decayed to background by the 24 h time point, in both the replication-defective WT (GNN) and WT conditions (Figure 1B, C, E, F and Supplementary Figures S1 and S2) (13,18). In contrast, the addition of exogenous miR-122, which is loaded into an Ago protein and interacts with the HCV genome at both site 1 and site 2, results in an ∼3.7-fold increase in luciferase activity in the establishment phase of the viral life cycle, quantified using the WT (GNN) condition at the 6 h time point, which we considered the initial impact on viral RNA stability and translation prior to viral RNA replication (Figure 1B, C and Supplementary Figure S1). During the maintenance phase of the viral life cycle, exogenous addition of miR-122 results in an approximately 17 487.2-fold increase in luciferase activity, quantified using the WT condition at the 72 h time point, after several rounds of viral RNA replication (Figure 1E, F and Supplementary Figure S2).

To further confirm that luciferase activity was an appropriate proxy for HCV reporter RNA levels, we performed strand-specific RT-qPCR to quantify viral RNA levels (Figure 1D, G). In contrast to our luciferase assay results, we observed similar viral RNA levels in the miR-122 and control miRNA conditions at early time points (2–8 h) post-electroporation for the WT (GNN) and WT experiment, while we observed significant differences in viral RNA accumulation at later time points (24–72 h) post-electroporation in the WT experiment (Figure 1D, G). The fold-change in viral RNA accumulation at the 72 h time point based on RT-qPCR analysis was approximately 410.6-fold (Figure 1G). This is consistent given that luciferase activity merely serves as a proxy for the proportion of viral RNAs engaged in translation and many luciferase proteins can be made from a single RNA molecule, while RT-qPCR is a more direct measure of total viral RNA levels. Additionally, it is possible that our RT-qPCR assay is not sensitive enough to detect the differences in HCV genome copy numbers, possibly due to the detection of 5′ to 3′ decay products.

Finally, to verify that our results were not an artifact of the miR-122 KO cells, we obtained a similar pattern when we performed the reciprocal experiment, whereby we made use of S1 + S2:p3 luciferase reporter HCV RNAs which contain point mutations in both miR-122 seed sequences, at site 1 (C26A) and site 2 (C41A). These specific point mutations were specifically chosen because they prevent endogenous miR-122 interactions, yet are predicted to maintain the overall secondary structure of the viral 5′ UTR (18). Ago:miR-122 interactions with sites 1 and 2 can then be rescued by exogenous complementation of miR-122 p3U molecules, which contain a compensatory mutation at position 3 of the miRNA (18). We introduced these S1 + S2:p3 GNN and S1 + S2:p3 viral RNAs into Huh-7.5 cells and complemented with miR-122 p3U or a control miRNA (Supplementary Figures S3-S5). Interestingly, we observed a lower overall fold change in both the luciferase activities, i.e. ∼2.7-fold (S1 + S2:p3 GNN) and ∼2211.4-fold (S1 + S2:p3), at the 6 h and 72 h time points post-electroporation, respectively; as well as a ∼14.5-fold change in HCV S1 + S2:p3 RNA copy number at the 72 h time point, when compared to the reciprocal experiment performed in miR-122 KO cells (Supplementary Figures S3–S5 versus Figure 1). While this could be due to slight changes in the folding of these two viral RNAs (i.e. WT versus S1 + S2:p3), or some intrinsic difference in the miR-122 KO cells acquired during selection, we believe that it is likely a result of competition for the RNA silencing machinery in Huh-7.5 cells, where endogenous miR-122 is highly abundant (∼16 000 copies/cell) (14).

Nonetheless, using a combination of these two assays we can estimate the overall contribution(s) of miR-122 complementation to both the establishment and maintenance phases of the viral life cycle. Overall, we found that full complementation of miR-122 at both site 1 and site 2 on the HCV genome provides an approximately 2.7- to 3.7-fold and 2211.4- to 17,487.2-fold increase in translation and viral RNA accumulation as measured by luciferase activity during the establishment and maintenance phases of the viral life cycle, respectively.

### miR-122’s riboswitch activity has a negligible impact on HCV translation and viral RNA accumulation

Next, we wanted to specifically dissect the precise contributions of each of the three roles attributed to miR-122 in the HCV life cycle, namely the: (i) riboswitch; (ii) genome stability and (iii) translational promotion activities. To do so, we used a combination of point mutations and compensatory mutant miR-122 molecules to manipulate miR-122 binding to site 1 and site 2 on the HCV genome.

**Figure 2.**
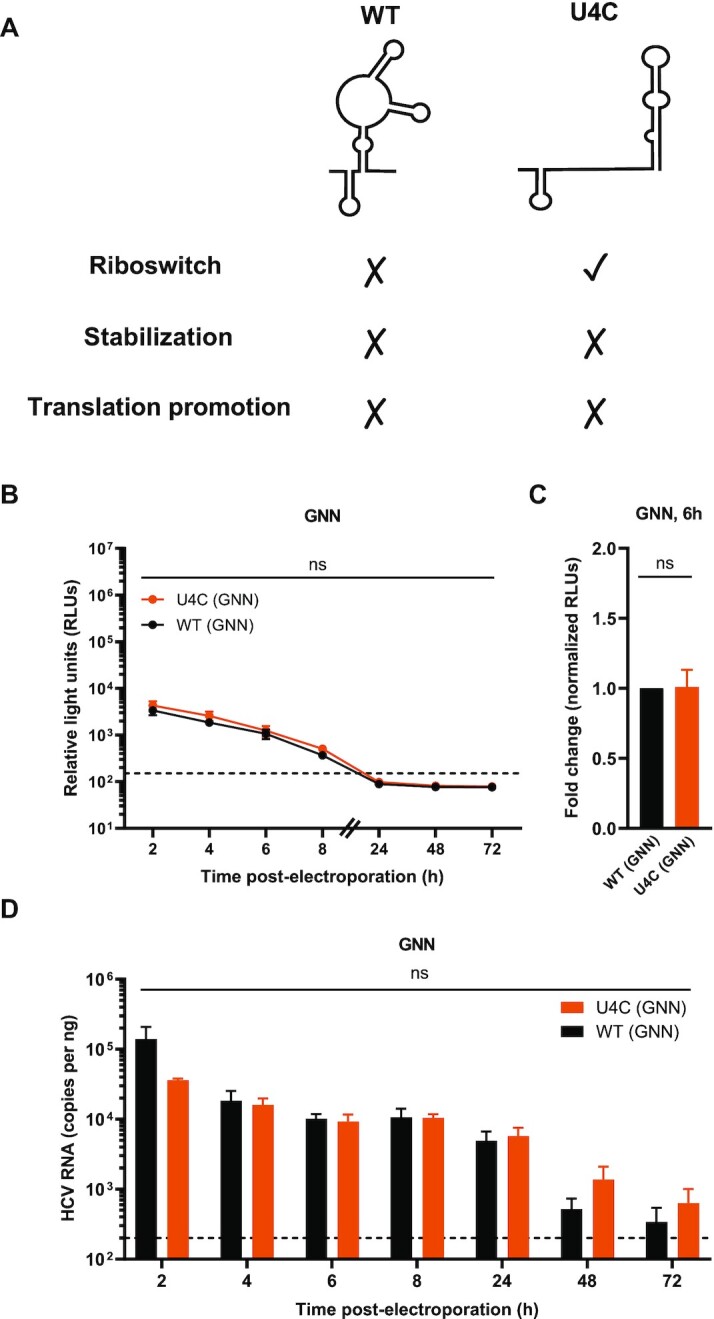
The riboswitch activity on its own has a negligible impact on viral translation. (**A**) Schematic representation of the experimental set-up for the riboswitch activity assay. In the absence of miR-122, WT HCV RNA favors the SLII^alt^ conformation, while the U4C mutant favors the ‘riboswitched’ (SLII) conformation. In the absence of miR-122, the stabilization and translational promotion roles of miR-122 are not fulfilled in either condition, allowing for isolation of the riboswitch effect. (**B**) Full-length RLuc WT (GNN) or U4C (GNN) HCV RNAs were co-electroporated into miR-122 KO cells with a capped FLuc reporter RNA. RLuc activity was monitored over time. The data in (B) is representative data from one of four independent biological replicates with three technical replicates, and error bars represent the standard error of the mean (SEM). The limit of detection is indicated. (**C**) RLuc activity at 6 h normalized to Fluc (transfection efficiency) control at 2 h, was used to calculate the fold change between WT (GNN) and U4C (GNN), with the WT (GNN) condition set to 1. Data is displayed as the mean of four independent biological replicates, and error bars represent the SEM. (**D**) Viral RNA levels were monitored by RT-qPCR as described in Figure 1. The limit of detection is indicated. Data is displayed as the mean of three independent biological replicates (except for the 8 h GNN and 6 h U4C GNN which represent two independent biological replicates), with error bars corresponding to the SEM. Statistical significance was determined by multiple Student's *t* test. ns, not significant (*P* ≥ 0.05).

Firstly, miR-122’s riboswitch activity is fulfilled when an Ago:miR-122 complex binds to site 2 on the HCV genome, converting the SLII^alt^ conformation to the SLII conformation (Figure 1A). However, miR-122 binding to site 2 also provides translational promotion via interactions between the Ago protein and SLII of the HCV IRES, which could conflate these two roles. As such, we decided to make use of the U4C mutant, which we previously demonstrated is ‘riboswitched’, even in the absence of miR-122 (Figure 2A and Supplementary Figures S6-7) (18). We compared WT (GNN) and U4C (GNN) HCV reporter RNA translation and viral RNA levels via luciferase assay in miR-122 KO cells. Since these viral RNAs do not replicate in the absence of miR-122 (Figure 2B-G), we only performed this experiment in the GNN (replication-defective) context (Figure 2B-D and Supplementary Figure S8) (18). Interestingly, we did not observe any significant differences in luciferase activity between WT (GNN) and U4C (GNN) at any time point post-electroporation, suggesting that the riboswitch effect has a minimal impact on HCV translation and viral RNA accumulation. We further quantified viral RNA levels using RT-qPCR and again observed no significant differences in viral RNA levels at any of the time points (Figure 2D). To confirm these results, we generated an additional mutant, G20A, which we demonstrate via *in vitro* selective 2′-hydroxyl acylation analyzed by primer extension (SHAPE) analysis is similarly ‘riboswitched’ in the absence of miR-122 (Supplementary Figures S6 and S7). In agreement with the U4C results, we did not observe any significant differences in luciferase activity or viral RNA levels between WT (GNN) and G20A (GNN) RNAs when electroporated into miR-122 KO cells (Supplementary Figures S9 and S10).

Given that the GNN reporter viral RNA produces luciferase even in the absence of miR-122 (Figures 1B, C and 2B, C), the viral 5′ UTR must be able to spontaneously ‘riboswitch’, even in the absence of miR-122. This is not surprising, given that the Gibb's free energies (ΔG) of the SLII^alt^ and SLII conformations are –39.7 and –37.8, respectively (Supplementary Figure S6). As such, the viral 5′ UTR likely exists in an equilibrium between these conformations within the host cell. Thus, although the riboswitch activity is required for viral translation, as it allows the HCV IRES to form, this is likely to be able to occur even in the absence of miR-122. Taken together, our results suggest that miR-122’s riboswitch activity has a negligible contribution to HCV translation and viral RNA accumulation, at least in the establishment phase of the HCV life cycle.

### miR-122-mediated genome stabilization plays a more important role in the establishment phase than in the maintenance phase of the viral life cycle

**Figure 3.**
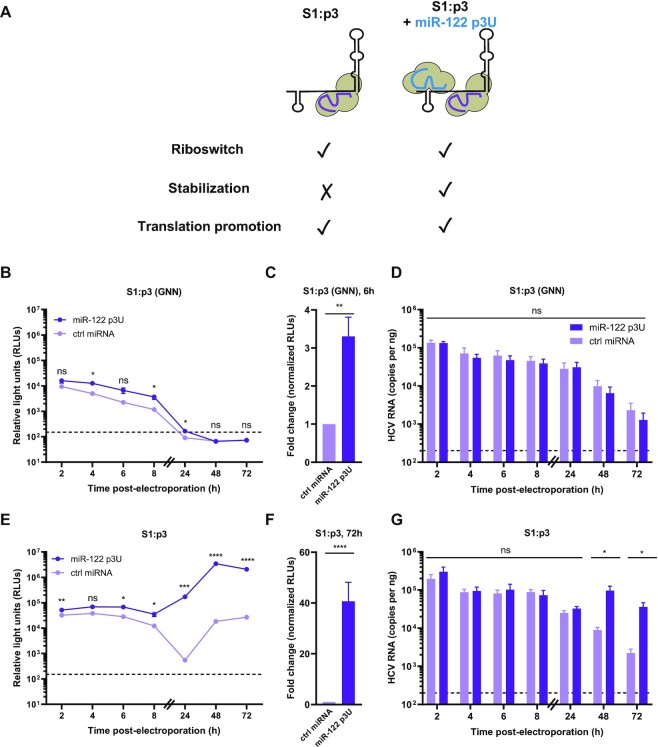
miR-122’s effect on genome stability has a greater impact in the establishment phase than in the maintenance phase of the HCV life cycle. (**A**) Schematic representation of the experimental set-up for genome stability assays. Endogenous miR-122 binds to site 2 only on HCV S1:p3 RNAs, thereby fulfilling the riboswitch and translational enhancement roles, while addition of miR-122 p3U allows binding to site 1 and measurement of the stabilization effect in isolation. (B–G) Full-length RLuc S1:p3 HCV RNAs were co-electroporated in Huh-7.5 cells with miR-122 p3U or ctrl miRNA, as well as a capped Fluc reporter RNA. Rluc activities for (B, C) S1:p3 (GNN) or (E, F) S1:p3 viral RNAs were monitored over time and is displayed as described in Figure 1. The limit of detection is indicated. Data in (**B**) and (**E**) are representative data from one of five independent biological replicates with three technical replicates, and error bars represent the standard error of the mean (SEM). In (**C**) and (**F**), the RLuc activity for S1:p3 (GNN) or S1:p3 HCV RNAs across all independent biological replicates at 6 h or 72 h, respectively, normalized to the FLuc (transfection efficiency) control at 2 h, were used to calculate the fold change, with the control miRNA condition set to 1. Data displayed is the mean of five independent replicates, and error bars represent the SEM. Viral RNA levels for (**D**) S1:p3 (GNN) and (**G**) S1:p3 were monitored by RT-qPCR (as described in Figure 1). The limit of detection is indicated. Data is displayed as the mean of four independent replicates with error bars corresponding to the SEM. Statistical significance was determined by multiple Student's *t* test, ****P≤ 0.0001; *** *P*≤ 0.001; **P ≤ 0.01; *P≤ 0.05 ns, not significant (*P* ≥ 0.05).

Next, we focused on miR-122’s role in genome stabilization, which is primarily mediated by the Ago:miR-122 interactions with site 1 through base pairing with the 5′ terminus of the viral RNA (Figure 1A) (16–19,22–24,37). As such, we made a point mutation in site 1 (C26A) in the HCV RNA, termed S1:p3, which prevents endogenous miR-122 interactions and can be rescued by exogenous complementation with miR-122 p3U molecules (Figure 3A) (18). To account for the riboswitch and translational promotion activities, endogenous miR-122 is available to interact with site 2 on the HCV genome, while the stability effect (primarily due to binding at site 1) can be complemented with compensatory miR-122 p3U molecules. S1:p3 (GNN) or S1:p3 HCV reporter RNAs were co-electroporated with miR-122 p3U or a control miRNA into Huh-7.5 cells and luciferase activity was monitored over time (Figure 3B, C and E, F and Supplementary Figures S11 and S12). In the establishment phase, we observed an ∼3.3-fold increase in luciferase activity upon complementation with miR-122 p3U (Figure 3B, C). However, in the maintenance phase, we observed an approximately 40.6-fold increase in luciferase activity upon miR-122 p3U complementation (Figure 3E, F). Again, we observed no significant differences in HCV RNA copies as measured by RT-qPCR during the establishment phase (Figure 3D). However, during the maintenance phase, we observed an approximately 16.2-fold increase in viral RNA levels in the miR-122 p3U-complemented compared to the control condition at the 72 h time point, in agreement with the luciferase assay results (Figure 3G).

To verify whether the accumulation of control miRNA-complemented S1:p3 in this experiment was real or due to reversion at site 1, we performed 5′ rapid amplification of cDNA ends (RACE) at the 72 h time point of the replication-competent experiment (Supplementary Figure S13). Of the 33 clones sequenced, 24 had 5′ truncations indicating active 5′-to-3′ decay, while 6 retained the S1:p3 (A26) mutation. However, two clones appeared to be a mixed population of WT (C26) and S1:p3, while one was WT. While we cannot confirm the identity of the S1:p3 position in the truncated clones, these results suggest that the majority of the viral RNAs retained the S1:p3 mutation, because WT revertants would be able to bind endogenous miR-122, and would be less vulnerable to 5′-to-3′ decay. Taken together, our results suggest that genome stabilization accounts for a significant proportion of miR-122’s overall effect during the establishment phase, but accounts for a lower proportion during the maintenance phase of infection.

### miR-122-mediated translational promotion is the predominant role in the maintenance phase of the viral life cycle

**Figure 4.**
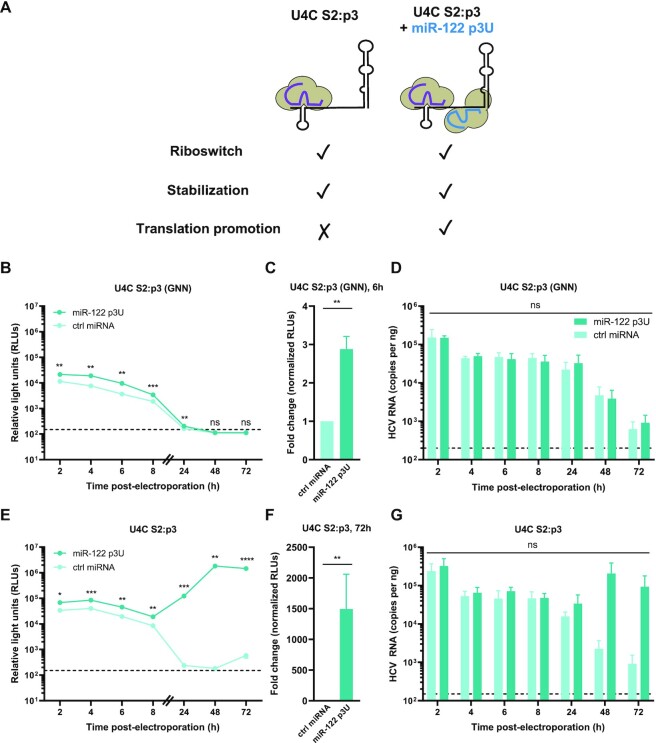
miR-122-mediated translational promotion is the dominant role during the maintenance phase of viral replication. (**A**) Schematic representation of the experimental set-up for translational promotion assays. HCV U4C S2:p3 is riboswitched *a priori*, and endogenous miR-122 can bind to site 1, thereby fulfilling the stabilization effect. Addition of miR-122 p3U allows binding to site 2 and measurement of the translational promotion effect in isolation. (B–G) Full-length RLuc U4C S2:p3 HCV RNAs were co-electroporated into Huh-7.5 cells with miR-122 p3U or ctrl miRNA, as well as a capped FLuc reporter RNA. RLuc activities for (**B**, **C**) U4C S2:p3 (GNN) or (**E**, **F**) U4C S2:p3 viral RNAs were monitored over time (as described in Figure 1). The limit of detection is indicated. Data in (B) and (E) are representative data from one of four independent biological replicates with three technical replicates, and error bars represent the standard error of the mean (SEM). In (C) and (F), the RLuc activity for U4C S2:p3 (GNN) or U4C S2:p3 HCV RNAs across all independent biological replicates at 6 or 72 h, respectively, normalized to the FLuc (transfection efficiency) control at 2 h, were used to calculate the fold change, with the control miRNA condition set to 1. The limit of detection is indicated. Data displayed is the mean of four independent biological replicates, and error bars represent the SEM. Viral RNA levels for (**D**) U4C S2:p3 (GNN) and (**G**) U4C S2:p3 were monitored by RT-qPCR (as described in Figure 1). The limit of detection is indicated. Data is displayed as the mean of three independent biological replicates with error bars corresponding to the SEM. Statistical significance was determined by multiple Student's *t* test, ****P ≤ 0.0001; *** *P*≤ 0.001; **P ≤ 0.01; *P ≤ 0.05; ns, not significant (*P* ≥ 0.05).

Finally, we focused on miR-122’s translational promotion effect, which is primarily mediated by contacts between the Ago protein bound to site 2 and the HCV IRES at SLII (Figure 1A). To measure the translational promotion effect in isolation, we introduced a point mutation at site 2 (C41A) in the HCV RNA, termed S2:p3, which prevents interactions with endogenous miR-122, but is predicted to maintain the overall secondary structure of the viral 5′ UTR, and can be rescued by exogenous complementation with miR-122 p3U molecules (Figure 4A) (18). To account for the riboswitch and genome stabilization activities, we also made use of the U4C point mutation (‘riboswitched’ *a priori*) and performed these experiments in Huh-7.5 cells, such that endogenous miR-122 can interact with site 1, providing the stabilization effect. We therefore co-electroporated U4C S2:p3 (GNN) or U4C S2:p3 HCV reporter RNAs with miR-122 p3U or a control miRNA into Huh-7.5 cells and monitored luciferase activity over time (Figure 4B, C and E, F and Supplementary Figures S14 and S15). In the establishment phase, we observed an ∼2.9-fold increase in luciferase activity upon addition of miR-122 p3U (Figure 4B, C). Interestingly, in the maintenance phase, we observed an ∼1493.8-fold increase in luciferase activity upon addition of miR-122 p3U, with luciferase counts only marginally above background in the control condition (Figure 4E-F). Again, we did not observe any significant differences in HCV RNA copies as measured by RT-qPCR during the establishment phase (Figure 4D). However, during the maintenance phase, we observed ∼100.0-fold higher viral RNA levels in the presence of miR-122 p3U compared to the control condition at the 72 h time point, in agreement with our luciferase results (Figure 4G). Notably, we observed a similar effect in the luciferase signal when this experiment was performed using G20A S2:p3 (GNN) and G20A S2:p3 viral RNAs, with an ∼21.0-fold difference in viral RNA levels as measured by RT-qPCR at the 72 h time point (Supplementary Figure S16–S18). Taken together, our results suggest that the translational promotion and genome stabilization effects have a similar overall contribution in the establishment phase of the viral life cycle, while in the maintenance phase translational promotion becomes the dominant role.

Given that the Ago protein was first identified as a ribosome-associated protein and it has been reported to contribute to miRNA-mediated translational promotion, we also wondered whether recruitment of Ago to the 5′ UTR of an unrelated transcript could lead to translational promotion (38–41). To investigate this, we used the Bacteriophage λN-BoxB system to directly tether human Ago2 to the 5′ end of a luciferase reporter mRNA (Supplementary Figure S19) (42). We transcribed the mRNAs using an ‘A cap’ [i.e. G(5′)ppp(5′)A] which stabilizes the transcript, but to prevent cap-dependent translation, since the eukaryotic initiation factor 4E (eIF4E) is not recruited to an A cap (43). As positive- and negative-controls, we used λN-eIF4G and λN-LacZ, respectively (Supplementary Figure S19A–C). In line with previous reports, recruitment of λN-Ago2 did not result in significant increases in luciferase activity beyond the negative control, suggesting that Ago2 does not universally promote translation when tethered to the 5′ end of a reporter mRNA (Supplementary Figure S19B) (11,12,15,26,31,44–47).

However, it remained possible that Ago2’s subsequent recruitment of the miRNA silencing effector protein, trinucleotide repeat-containing gene 6 (TNRC6), could affect Ago2’s translational promotion activity. As such, we also engineered point mutations which disrupt the Tryptophan (Trp)-binding pockets in Ago2 that mediate TNRC6 recruitment. Of the three TNRC6 Trp-binding pockets (termed 1, 2 and 3) present on Ago2, we generated all single and double mutants, and then tested them in the same tethering assay (48–50). We did not include a triple Trp-binding pocket mutant, as this mutant is unstable (Ian MacRae, personal communication). In all cases, we did not observe translational promotion by any of the Ago2 Trp-binding pocket mutants, suggesting that even when TNRC6 recruitment is impaired, Ago2 is still not able to promote cap-independent translation when directly tethered to the 5′ UTR of a target mRNA (Supplementary Figure S19C). Thus, in line with previous studies, Ago-mediated translational promotion is context-dependent (11,12,15,26,31, 44–47).

### The SLII^alt^ conformation is likely maintained because it promotes efficient virion assembly

**Figure 5.**
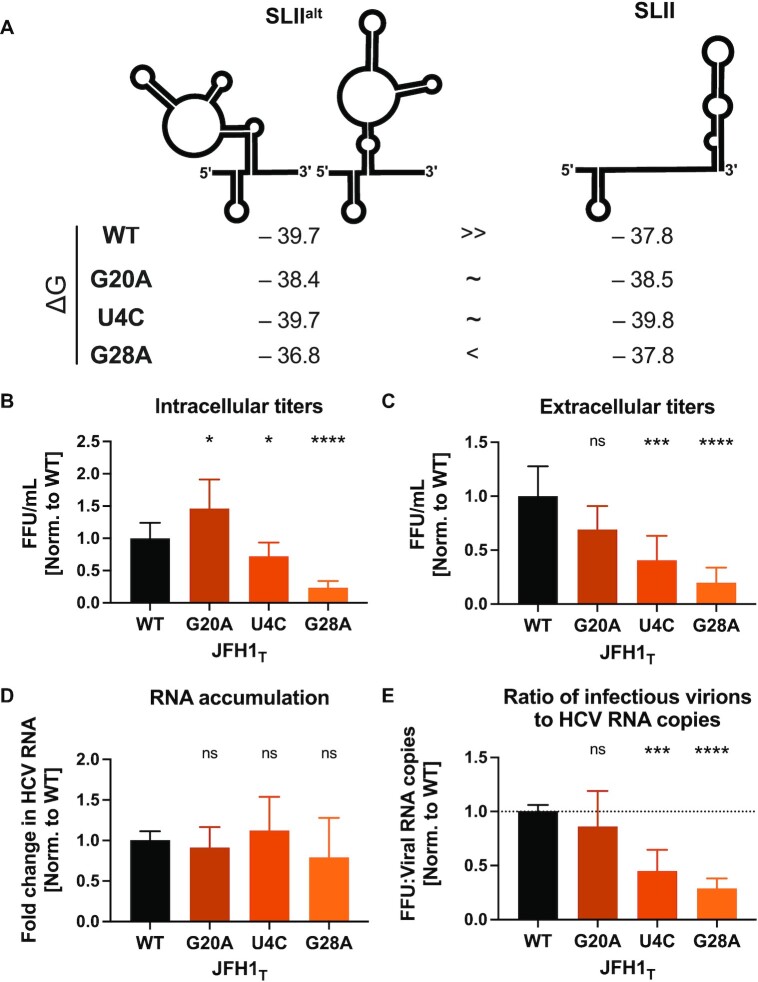
Successive stabilization of the SLII conformation leads to a reduction in viral particle production. (**A**) RNA conformations of the first 117 nucleotides of the HCV 5′ UTR, showing the Gibb's free energies (Δ*G*) of SLII^alt^ and SLII for WT, G20A, U4C and G28A RNAs. Three days post-electroporation of JFH-1_T_ RNA with the indicated mutations into Huh-7.5 cells, (**B**) intracellular viral titers and (**C**) extracellular viral titers were assessed by focus-forming unit (FFU) assay, and (**D**) viral RNA accumulation was assessed by RT-qPCR. (**E**) The proportion of packaged RNAs was ascertained by calculating the ratio of total FFUs (A, B) to viral genome copies (C). The relative titers, RNA levels, and percentages of packaged virions (A–D) were all normalized to the WT condition. All data displayed represent the mean of at least three independent biological replicates and error bars represent the SD of the mean. Statistical significance was determined by multiple Student's *t* test, ****P < 0.0001, ***P < 0.001; **P < 0.01; ns, not significant (*P* ≥ 0.05).

In our efforts to understand the contribution of miR-122’s riboswitch, genome stabilization, and translational promotion activities, we were somewhat surprised by the negligible contribution we observed for the riboswitch effect. While it is clear that the viral RNA can spontaneously riboswitch even in the absence of miR-122, we questioned why HCV would retain the need to riboswitch at all (i.e. why conserve the ability to form SLII^alt^ over SLII?). To test this, we made use of point mutations in the 5′ UTR that stabilize the SLII conformation, specifically G20A, U4C, and G28A (Figure 5A and Supplementary Figure S6) (18,19). The U4C and G20A mutations change the U-G wobble base pair at the base of SLI into a C-G (U4C) or U-A (G20A) Watson-Crick base pair, which results in similar energetic stabilities (ΔG) for the SLII^alt^ and SLII conformations. Meanwhile, the G28A mutation destabilizes SLII^alt^, rendering the SLII conformation comparatively more stable (17–20).

Since we did not observe a significant effect of the riboswitch activity in the establishment phase of the viral life cycle, and because the large luciferase reporter insertion impairs virion production, we decided to test these mutants in the context of the JFH-1_T_ HCV cell culture (HCVcc) system (30,32,51,52). Thus, we introduced each of the point mutations into the JFH-1_T_ genome, and electroporated these viral RNAs into Huh-7.5 cells (which are replete with endogenous miR-122), then monitored viral RNA accumulation, as well as intracellular and extracellular viral titers (Figure 5B–E). Fascinatingly, despite similar amounts of viral RNA accumulation, we noticed an inverse correlation between the relative stability of SLII and the intracellular and extracellular viral titers at day 3 post-electroporation. Notably, U4C and G28A consistently packaged a smaller proportion of viral genomes than WT or G20A (Figure 5E). These results suggest that the SLII^alt^ conformation is likely retained as it is required for efficient packaging of the genomic RNA into virions.

## DISCUSSION

Herein, we investigated the overall contribution of each of the three roles attributed to miR-122 in the HCV life cycle – namely riboswitch activity, genome stabilization, and translational promotion. In general, we found that the relative contributions of the three roles differed during the establishment and maintenance phases of the viral life cycle. First, we used replication-defective (GNN) viral RNAs to estimate the contribution of each of the three roles to miR-122’s activity in the establishment phase of the HCV life cycle (i.e. prior to the initiation of replication). While we observed that the riboswitch effect played a negligible role during this phase, we know that the genome must be ‘riboswitched’ for translation, as the SLII conformation forms part of the IRES (SLII-IV). Thus, while WT HCV can spontaneously switch conformations due to the similar thermodynamic stabilities between the SLII^alt^ and SLII conformations (Supplementary Figure S6), the effect of stabilizing the SLII conformation is likely below the limit of detection in our assay. Additionally, we found that during the establishment phase, the contributions of the genome stability and translational promotion effects were similar in magnitude. Notably, at the 6 h time point used for quantification, we observed that both the replication-defective (GNN) and replication-competent luciferase values were similar in magnitude and fold-change, suggesting that no significant viral replication has occurred at this time point (Figures 1-4).

In contrast, using replication competent viral RNAs, we were able to estimate the contribution of each of the three roles to miR-122’s activity in the maintenance phase of the HCV life cycle (i.e. after several rounds of viral replication). Unlike the establishment phase, where the genome stability and translational promotion effects had similar overall contributions, we found that translational promotion was the predominant role accounting for the vast majority of miR-122’s activity during the maintenance phase of the viral life cycle (Figure 4).

These results align with the current understanding of the HCV life cycle, given that in the early phases of infection, prior to the establishment of viral RNA replication, each HCV RNA molecule must be stable long enough and produce sufficient levels of viral proteins to establish a replication organelle. However, once a replication organelle is established, the negative-strand replicative intermediate can produce many positive-sense progeny genomic RNA molecules. As such, viral RNA stability becomes less important, while generating sufficient levels of viral proteins to establish subsequent replication organelles becomes the limiting factor. This is consistent with previous observations that suggest that miR-122-independent replication is possible, albeit highly inefficient, when HCV nonstructural protein translation is driven by the more efficient encephalomyocarditis virus (EMCV) IRES (22,53). This is also in agreement with recent studies that similarly explored the contributions of the riboswitch, genome stability and translational promotion activities, and proposed that miR-122’s impact is greatest before 72 h post-electroporation because it promotes the synthesis of enough viral proteins to allow the establishment of replication organelles prior to degradation of the input viral RNAs (53,54).

Interestingly, several previous studies which attempted to discriminate the different roles of miR-122 have come to differing conclusions, which may be due at least in part by the different systems used to study the different roles (15,16,22,25,26,53–55). For example, one study pointed to a role for miR-122 in promoting viral RNA synthesis prior to translation (55). However, our results and that of others, showing increased translation of replication-defective (GNN) RNA upon miR-122 addition, suggest that miR-122’s role in translational promotion is distinct from that on viral RNA synthesis (54). Although some of the effect on the GNN construct may be attributable to miR-122-mediated genome stabilization, our observation that site 2 binding results in increased luciferase activity for a viral genome that is ‘riboswitched’ *a priori*, and is bound by miR-122 at site 1, supports this translational promotion effect, given site 1-bound miR-122 is the main contributor to genome stabilization (16,18,21,37,56). However, it is possible that Ago:miR-122 interactions with site 2 also contribute to some extent to genome stabilization (22,25,37,46).

Early studies examining miR-122-mediated translational promotion also suggested that both miR-122 binding sites were important in IRES-mediated translation, since reductions in translation were observed upon mutation of either site (15,26). However, according to the current model (Figure 1A), binding to site 1 indirectly affects translation by triggering the shift in the site 2-bound Ago's positioning, promoting further interactions with the HCV IRES (18). Additionally, prior to identification of the SLII^alt^ and SLII conformations of the HCV 5′ UTR, several studies made use of alternative miR-122 seed site point mutations, which in hindsight are predicted to alter the conformations of the HCV 5′ UTR in a manner that may have conflated the importance of the two miR-122 binding sites (11–13,16,19,20,22,25,26,55,57,58). For example, RNA structure prediction analyses suggest that the S1:p6U mutant (A23U, based on JFH-1 nucleotide sequence) adopts the riboswitched conformation *a priori*; while the S2:p3G (C26G) mutant is not predicted to preferentially form the functional SLII conformation in the absence of miR-122. As such, the S1:p3A (C26A) and S2:p3A (C41A) mutations used herein were carefully selected due to their minimal perturbations to the predicted conformation(s) of the viral RNA (18). Moreover, the use of the U4C S2:p3U mutant in Huh-7.5 cells in our translation assays, allows us to better isolate the effect of miR-122 on translation without confounding the indirect effects of site 1 binding. Thus, while our results are largely in agreement with previous studies, the discrepancies are likely attributable to the different systems and/or mutations made in the miR-122 binding sites.

In addition to assessing the contribution of each of the three roles in the viral life cycle, we also wanted to further explore the role of Ago in promoting translation, as it was initially identified as a ribosome-associated factor and has been reported to be important in miRNA-mediated translational promotion of AU-rich element containing mRNAs during stress (38,40). Moreover, a recent study has extended this phenomenon to Ago-interactions with mRNAs that are upregulated in cancer cells (41). In both cases, Ago was found to be required for efficient miRNA-mediated translational promotion, and Ago tethering experiments suggested a function independent of miRNA interactions (38). Thus, to explore this further, we investigated whether recruitment of Ago to the 5′ UTR of an unrelated transcript would result in cap-independent translational promotion, like what we observe during HCV infection. While our results did not reveal any Ago-mediated translational promotion, we were curious whether Ago-mediated translational promotion may be masked by downstream recruitment of the miRNA silencing effector protein, TNRC6 (59–62). Thus, we engineered point mutations to disrupt the Trp-binding pockets in Ago, thereby interfering with Ago's ability to recruit TNRC6 (48,49). In agreement with previous studies which tethered Ago to the 3′ UTR of reporter RNAs, we did not observe Ago-mediated translational promotion, suggesting that Ago-mediated translational promotion is indeed context dependent (63).

**Figure 6.**
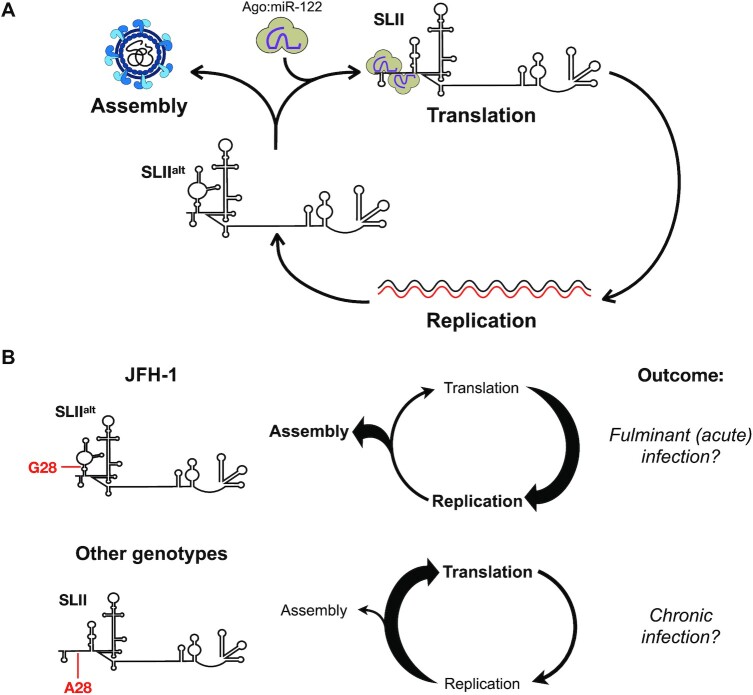
Model for the functional role of SLII^alt^ in the HCV life cycle. (**A**) Newly synthesized viral genomes are at a junction point in the HCV life cycle: they can either engage in virion assembly and be packaged into new virions that exit the cell, or they can engage in a new cycle of translation and viral RNA replication. Our model suggests that newly synthesized RNAs that take on the most energetically stable conformation, SLII^alt^, can engage in packaging. However, if they spontaneously, or through binding to Ago:miR-122, convert to the functional SLII conformation, they form the viral IRES and recruit ribosomes, committing to translation. (**B**) Previous studies indicate that JFH-1 nonstructural proteins provide it with a replicative advantage over other HCV isolates. Additionally, the G28 residue in the 5′ UTR allows JFH-1 (and likely other genotype 2 isolates) to energetically favor the SLII^alt^ conformation, which we demonstrate promotes virion assembly. This prioritization of virion assembly over translation may contribute to JFH-1 pathogenesis, considering it was isolated from a patient with fulminant (acute) hepatitis and genotype 2 has been associated with increased incidence of fulminant hepatitis. In contrast, other HCV genotypes (∼80% of all HCV isolates) have an A at position 28 (A28), which energetically favors the SLII conformation, rather than SLII^alt^. As a result, these other genotypes favor translation over virion assembly. Moreover, A28 increases the affinity of the 5′ UTR for Ago:miR-122 complexes, further reinforcing translation. This bias may allow the virus to better evade intracellular and systemic immune responses, thereby resulting in chronic HCV infection.

Finally, while the negligible contribution of the riboswitch effect can be explained by the similar thermodynamic stabilities of the SLII^alt^ and SLII conformations in the 5′ UTR of the HCV genome, our experiments using the HCVcc system revealed that the SLII^alt^ conformation is also important for efficient virion assembly. This conclusion is based on our observation that mutations that stabilized SLII over SLII^alt^ reduced viral packaging efficiency of HCVcc. More specifically, without significantly altering intracellular viral RNA accumulation as measured by RT-qPCR analyses, we found that the U4C and G28A mutations significantly reduced both intracellular and extracellular viral titers. The latter is consistent with a prior study, which reported that a G28A virus produced significantly lower infectious viral progeny than WT HCVcc, even in miR-122-replete conditions (28). Herein, we also found that U4C, which stabilizes SLII without significantly destabilizing SLII^alt^, reduced virion production but to a lesser extent than G28A. Interestingly, the G20A mutation did not have a significant impact on virion production, even though its secondary structure closely matches that of U4C. Notably, both U4C and G20A stabilize the SLII conformation by replacing a G-U wobble with a canonical Watson-Crick base pair at the base of SLI. G20A generates a U-A base pair, which has a similar stability to the G-U wobble, while U4C creates a C-G base pair, which is thermodynamically more stable (64). Thus, although U4C or G20A are similarly likely to form the SLII^alt^ or SLII conformations, the G20A mutation results in a similar thermodynamic stability to WT and thus it may simply be more dynamic (Supplementary Figure S6). Nonetheless, our results suggest a model whereby the SLII^alt^ conformation promotes virion assembly, while the SLII conformation promotes viral translation (Figure 6A). Given that miR-122 is not packaged into HCV virions, it is likely that miR-122 interactions would reinforce the commitment of progeny genomes into translation, whereas the SLII^alt^ conformation facilitates their commitment to virion assembly (65).

Interestingly, our results may also help explain the ability of the JFH-1 strain to produce infectious particles in cell culture (Figure 6B). The JFH-1 strain (genotype 2a) was the first infectious HCV isolate to recapitulate the entire HCV life cycle in cell culture without adaptive mutations, including viral particle production (66–69). Subsequently, chimeric viruses that combine the JFH-1 genome with core-NS2 or 5′ UTR-NS2 sequences derived from genotypes 1–6 were developed and shown to produce infectious viral particles in cell culture (reviewed in (70)). While the ability to produce infectious virions is certainly due at least in part to the JFH-1 nonstructural genes, our findings herein suggest that there may also be a contribution from the G residue at position 28 (G28) in the 5′ UTR. Notably, ∼80% of all HCV isolates contain an A at this position (A28) (71). In JFH-1, the presence of G28 provides thermodynamic stability to SLII^alt^, and as we demonstrate herein, helps to prioritize virion assembly (Figure 6B) (72–74). In contrast, in other HCV genotypes, the A28 residue stabilizes the SLII conformation, which promotes viral translation to the detriment of virion assembly (Figure 6B). Moreover, the A28 residue also increases the affinity of the viral RNA for Ago:miR-122 at both miR-122 binding sites, which likely further reinforces a commitment to translation (18,49,71,75). Taken together, this suggests that while JFH-1 can prioritize virion assembly over translation, the other genotypes end up lost in translation.

Lastly, it is interesting to consider whether our findings may also help explain HCV infection outcomes. Notably, JFH-1 was isolated from a patient with fulminant (acute) hepatitis, and several case reports have suggested an association between genotype 2 and fulminant hepatitis or severe recurrence post-transplant (67,76,77). However, this association may be related to higher incidence of genotype 2 in these patient populations, as fulminant hepatitis cases have also been reported with other genotypes (78–80). Moreover, despite its worldwide distribution, genotype 2 (G28) only accounts for an estimated 9.1% of HCV cases globally, and is highly responsive to interferon-free direct-acting antiviral regimens (80–82). It is unclear if the lower prevalence of genotype 2 is related to a higher rate of spontaneous viral clearance or to other factors, as genotype 2 has been sporadically reported to be less likely to progress to chronic infection — with estimates between 22–33% for genotype 2 versus 57–92% for genotype 1 (82,83). However, spontaneous clearance rates between genotypes are unreliable, as they largely rely on the assessment of outbreak studies of single genotypes in distinct patient populations and are thus subject to cohort bias (81–83). Nonetheless, it is possible that more efficient replication and infectious particle production generates a more robust intracellular and systemic immune response that can effectively control HCV infection at the acute stage. In contrast, prioritizing translation to the detriment of virion assembly may allow HCV to better evade intracellular antiviral responses and avoid induction of a systemic immune response, thereby favoring the establishment of a chronic infection. Consistent with this idea, treatment-naïve chronic genotype 2 patients have been demonstrated to have greater HCV-specific T cell responses at baseline, which could be reflective of early viral infection kinetics (84). However, more research is needed to provide further insight into whether G28 status influences disease pathogenesis.

In conclusion, our results help clarify the overall contributions of the three roles attributed to miR-122 in the HCV life cycle, namely the riboswitch, genome stability and translational promotion activities. During the establishment phase of the viral life cycle, miR-122’s stabilization and translational promotion activities each contribute to a similar extent to the overall impact on viral translation and RNA accumulation, suggesting that a balance between these two functions is important for optimal establishment of an infection. In the maintenance phase, once replication complexes have been established, the contribution of the stabilization effect represents a minor fraction of the overall impact, and the translation promotion effect becomes the dominant role. Moreover, while we found that the riboswitch effect had a negligible impact on viral translation and RNA accumulation, the ability of the viral genomic RNA to form the SLII^alt^ conformation was important for efficient virion assembly. Our data suggest that the ability to form SLII^alt^ may partially explain infectious particle production in HCVcc models and may offer insight into the outcome of HCV infection. Overall, our results help clarify the importance of miR-122 as well as SLII^alt^ in modulating the balance of viral RNAs in the translating/replicating pool and those engaged in virion assembly.

## DATA AVAILABILITY

The data underlying this article will be shared on reasonable request to the corresponding author.

## Supplementary Material

gkad094_Supplemental_File
