# Supplementary material for: Elucidating the distinct contributions of miR-122 in the HCV life cycle reveals insights into virion assembly
Source: Nucleic Acids Res. 2023 Feb 21;51(5):2447–63. doi: 10.1093/nar/gkad094 (PMC10018354; doi:10.1093/nar/gkad094)
Supplement: gkad094_Supplemental_File [file gkad094_supplemental_file.pdf]

Supplementary Materials

Supplementary Methods

Cell culture

HEK 293T human embryonic kidney cells were obtained from the American Type Culture Collection (ATCC) and maintained in Dulbecco's minimal essential medium (DMEM) supplemented with 10% fetal bovine serum, 1X MEM nonessential amino acids, and 2 mM L-glutamine. Cells were maintained at 37°C/5% CO_2 and were routinely screened for mycoplasma contamination.

Plasmids

The plasmid pMSCV-LN-eIF4G encoding a FLAG-tagged Bacteriophage Lambda N peptide (λN) fused eIF4G protein as well as the plasmids containing the 6X BoxB and 6X scrambled (scr) sequences were provided by Dr. Jerry Pelletier (McGill University). The pcDNA3.1+Lambda-N-HA-Peptide (Addgene plasmid # 92005; http://n2t.net/addgene:92005 ; RRID:Addgene_92005) and pcDNA3.1+_FH-AGO2-WT (Addgene plasmid #92006 ; http://n2t.net/addgene:92006 ; RRID:Addgene_92006) were gifts from Dr. Joshua T. Mendell (1). The plasmid pCDNA3.1+LN-FLAG-HA-LacZ encodes a FLAG- and HA-tagged λN fused LacZ. The plasmid pcDNA3.1+LN-FLAG-HA-Ago2 encoding a FLAG-tagged λN fused human Ago2, was generated using the In-Fusion cloning kit (Takara Bio) by PCR amplification of the Ago2 gene from the pcDNA3.1+_FH-AGO2-WT plasmid and insertion into the *XhoI* digested pcDNA3.1+Lambda-N-HA-Peptide plasmid by Gibson assembly according to the manufacturer's instructions. The Trp-binding pocket mutations (K660S, P590G and R688S) were introduced into the pcDNA3.1+LN-FLAG-HA-Ago2 by site-directed mutagenesis using

the QuikChange XL II kit (Agilent) according to manufacturer's instructions. All plasmid sequences were verified by Sanger sequencing (Génome Québec).

In Vitro Transcription

To make A-capped 6X BoxB and 6X scrambled (scr) mRNAs, the plasmids were linearized with *BamHI* and *in vitro* transcribed using T7 RNA polymerase (NEB). Briefly, 1 µg of template DNA was incubated at 37°C for 1 h with 500 µM each G(5´)ppp(5´)A cap analog (NEB), ATP, CTP and UTP, 100 µM GTP, 0.2 U/µL RiboLock RNase inhibitor and 250 U T7 RNA polymerase in a final volume of 100 µL, followed by a 20 min DNase I (NEB) digest at 37°C. Capped RLuc mRNAs were generated from the pRL-TK plasmid (Promega) linearized with *BglII* and were *in vitro* transcribed using the mMESSAGE mMACHINE T7 Transcription Kit (Invitrogen) according to the manufacturer's instructions. *In vitro* transcribed RNAs were precipitated in 0.1 volume of 3M NaOAc pH 5.2 and 2.5 volumes 95-100% ethanol with 1 µL GlycoBlue^{TM} co-precipitant (ThermoFisher Scientific) and stored at -80°C until use.

Tethering Assays

For hAgo2 tethering assays, 1 × 10^6 HEK 293T cells were seeded in each well of three 6-well plates approximately 16 h prior to transfection. Subsequently, 3 µg of pMSCV-LN-eIF4G or pcDNA3.1+LN-FLAG-HA-Ago2 (WT as well as single and double Trp-binding pocket mutants), or 1 µg pCDNA3.1+LN-FLAG-HA-LacZ plasmid were transfected using 2.7 µL Lipofectamine 2000 (ThermoFisher Scientific) per well in 1 mL of Opti-MEM reduced serum media (ThermoFisher Scientific) in duplicate. Transfection complexes were replaced with complete media at 3.5 to 4 h post-transfection. At 24 h post-transfection, the cells were split into

12-well plates and 5-6 h later were transfected with 400 ng each A capped [A(5′)ppp(5′)G] 6X BoxB or 6X scr mRNAs and RLuc control mRNA using 2 µL DMRIE-C reagent (ThermoFisher Scientific) in 400 µL Opti-MEM per well. Transfection complexes were left on the cells overnight, and cells were harvested in 100 µL 1X PLB 16 h post-transfection. Experiments were conducted in three independent replicates with two technical replicates (two wells) per condition within each experiment.

Western Blot Analysis

Whole cell lysates were prepared in 1X passive lysis buffer (Promega) and stored at -80 °C until use. Lysates were cleared by centrifugation at 16,000 × g and supernatant protein concentration was assessed by Bradford assay using the Pierce Coomassie assay kit (ThermoFisher Scientific) according to manufacturer's instructions, with the modification that 1-2 µL of supernatant and 5 µL of standard was used with 200 µL of Coomassie protein assay reagent. Ten micrograms of protein was loaded on 10% SDS-PAGE gels and run at 80 V for 20 min, followed by 100V. Samples were transferred onto Immobilon-P PVDF membranes (Millipore), blocked in 5% skim milk for 1 h and incubated overnight with the following primary antibodies diluted in 5% bovine serum albumin (BSA): mouse anti-FLAG M2-horse radish peroxidase (HRP) (F8592, Sigma, 1:20,000 or 1:200,000) and rabbit anti-actin (A2066, Sigma, 1:20,000) When probing for actin, blots were washed in Tris buffered saline with Tween 20 (TBS-T) and incubated for 1 h with HRP-conjugated goat anti-rabbit (111-035-144, Jackson ImmunoResearch Laboratories, 1:50,000). After washing, blots were visualized using enhanced chemiluminescence (ECL Prime Western Blotting Detection Reagent, Fisher Scientific).

***In Vitro* Selective 2′ Hydroxyl Acylation Analyzed by Primer Extension (SHAPE)**

In vitro SHAPE analysis was performed in quadruplicate as previously described (2). Briefly, 5 pmol of HCV 5′ UTR RNA was re-folded and incubated in SHAPE buffer (333 mM HEPES, pH 8.0; 20 mM $MgCl_2$; 333 mM NaCl) for 1 h at 37ºC. RNA was then exposed to 0.01 M NAI-N_3 or dimethyl sulfoxide (DMSO) (treatment control) for 5 min at 37ºC, and then extracted using TRIzol reagent (ThermoFisher Scientific) according to the manufacturer's instructions. Extracted labelled RNA was precipitated and stored at -80ºC. Labelled RNA was used for SHAPE analysis by capillary electrophoresis as previously described (3).

5′ Rapid Amplification of cDNA Ends (RACE)

Reverse transcription. Huh-7.5 cells from each well of a 6-well plate were lysed in 500 µL TRIzol reagent (ThermoFisher Scientific). Samples were stored at -80°C until use and RNA was extracted using chloroform, according to the manufacturer's instructions. Reverse transcription of the 5′ end of the genome was performed using the HCV-specific primer HCV_RACE_RT (5′ – CGC GCG GTC CGC CGG GTA GAA TTC/iSp18/GCC CGG AAA CTT AAC GTC TT – 3′) which had previously been 5′ phosphorylated with T4 polynucleotide kinase (T4 PNK, NEB) according to manufacturer's instructions, with the modification that the 37°C incubation was increased to 1 h. Briefly, 1 µg total RNA was incubated with 100 nM monophosphorylated primer and dNTP mix (1 mM each) at 95°C for 5 min to remove any secondary structure. The reaction mixture was then placed on ice and First Strand buffer, DTT to 10 µM, 20 U RiboLock RNase inhibitor and 200 U SuperScript III reverse transcriptase were added. The mixture was then incubated at 53°C for 30 min to allow for cDNA synthesis, and the enzyme was heat inactivated at 70°C for 15 min. Eight microliters of a 0.5 N NaOH/0.25 M EDTA solution was added, and the reaction mixture was

heated to 65°C for 15 min to hydrolyze the template RNA. Water was added to 200 μL total and the cDNA was then purified by ethanol precipitation.

cDNA circularization. After ethanol precipitation, the cDNA was resuspended in 7.3 μL water. One microliter of DMSO was added and the mixture was heated at 95°C for 5 min to denature secondary structures, followed by a 1 min incubation on ice and quick centrifugation. The following components were added as a master mix: T4 RNA ligase buffer, 1 μM ATP, 22.5% PEG 8000, 5 U T4 RNA ligase 1 (high concentration, NEB). The reaction mixture was mixed by gentle pipetting, and an additional 10 U of T4 RNA ligase 1 was added, for a total volume of 20 μL. The reaction was incubated at room temperature overnight, with gentle pipetting every 30 min for approximately 2-3 h at the onset of the reaction.

PCR amplification. Two T4 RNA ligase reactions were combined and ethanol precipitated, and the circularized cDNA was resuspended in 10 μL water. The circular cDNA was then amplified using the Q5 Hot Start High-Fidelity 2X Master Mix (NEB) with the primers HCV_RACE_PCR_FOR (5′ – GAA TTC TAC CCG GCG GAC CGC GCG – 3′) and HCV_RACE_PCR_REV (5′ – TCT AGA CGG TCT ACG AGA CCT CCC GGG GC – 3′). The PCR product was purified by gel extraction from a 1.5% agarose gel using the QIAquick gel extraction kit (Qiagen) according to manufacturer's instructions.

Gibson assembly reaction. The pUC18 vector was generated by amplification of a pUC18_ZIKV plasmid with the primers pUC18_ZIKV_RACE_FWD (5′ – AGG TCT CGT AGA CCG GGG TCG GCA TGG CAT CTC – 3′) and pUC18_ZIKV_RACE_REV (5′ – GCG GTC CGC CGG GTA GAA TTC GTA ATC ATG GTC ATA GCT GTT TC – 3′). This vector was gel extracted, treated with *DpnI* (NEB) for 1 h to remove template plasmid DNA, and purified using the QIAquick PCR purification kit (Qiagen) according to manufacturer's instructions. The insert

generated by PCR amplification of the circular cDNA was combined in a 2:1 ratio with the vector and ligated by Gibson assembly using the In-Fusion HD Cloning Kit (Takara Bio) according to manufacturer's instructions.

Sequencing. The Gibson assembly ligation reaction was transformed into NEB stable or 10 beta cells and at least 10 colonies were screened per experimental replicate (33 colonies total). Plasmid DNA was purified using the QIAprep spin miniprep kit (Qiagen) according to manufacturer's instructions, and Sanger sequencing was performed at Génome Québec.

References

1. Golden, R.J., Chen, B., Li, T., Braun, J., Manjunath, H., Chen, X., Wu, J., Schmid, V., Chang, T.C., Kopp, F. *et al.* (2017) An Argonaute phosphorylation cycle promotes microRNA-mediated silencing. *Nature*, **542**, 197-202.
2. Chahal, J., Gebert, L.F.R., Camargo, C., MacRae, I.J. and Sagan, S.M. (2021) miR-122-based therapies select for three distinct resistance mechanisms based on alterations in RNA structure. *Proc Natl Acad Sci U S A*, **118**.
3. Chahal, J., Gebert, L.F.R., Gan, H.H., Camacho, E., Gunsalus, K.C., MacRae, I.J. and Sagan, S.M. (2019) miR-122 and Ago interactions with the HCV genome alter the structure of the viral 5' terminus. *Nucleic Acids Res*, **47**, 5307-5324.

Supplementary Figures

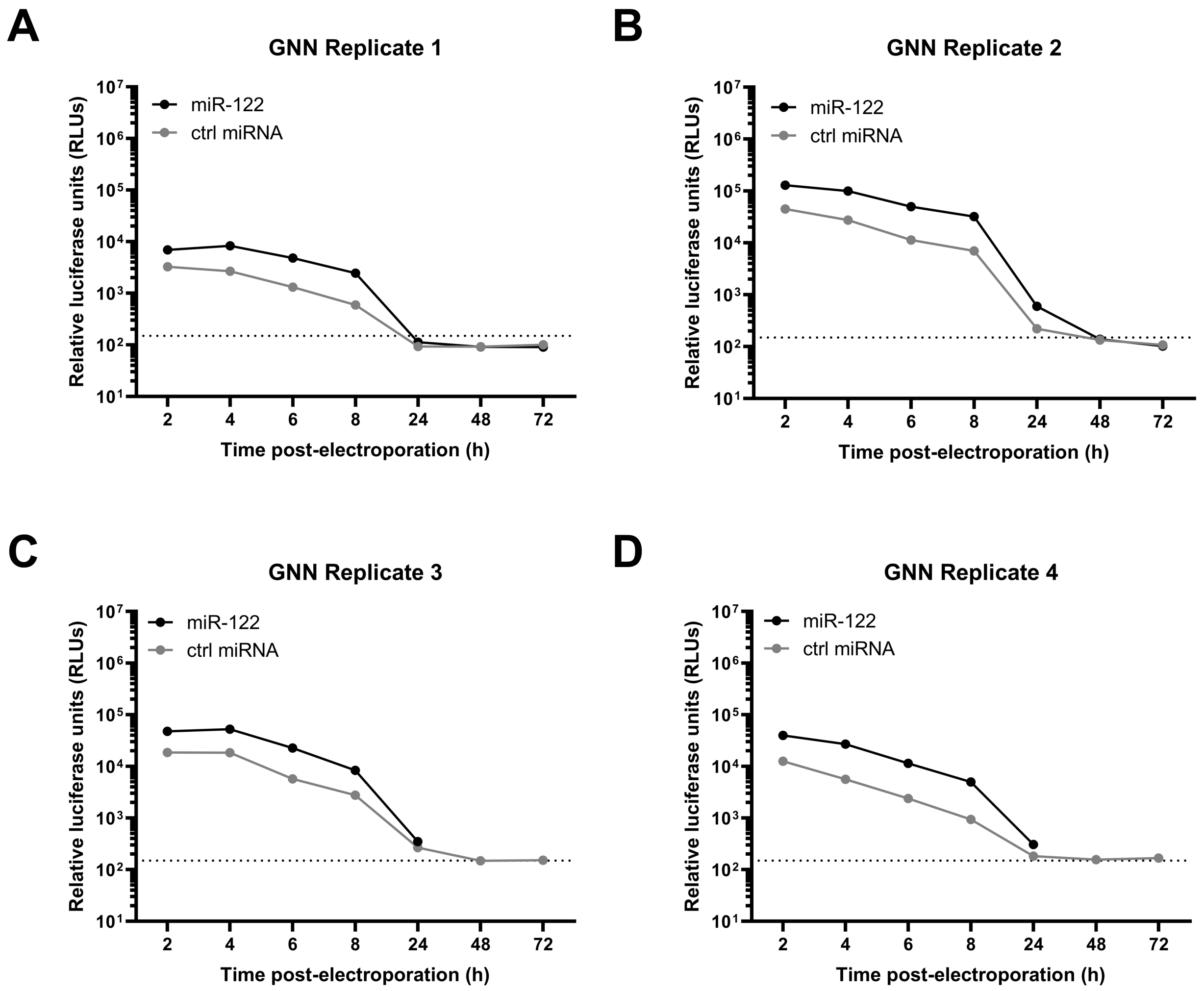

Figure S1. Independent biological replicate (GNN) data for the overall contributions of miR-122's riboswitch, genome stability, and translation promotion activities. (A-D) Full-length RLuc HCV (GNN) RNAs were co-electroporated into miR-122 knockout (KO) cells with miR-122 or control (ctrl) miRNA, and a capped Firefly luciferase (FLuc) reporter RNA. RLuc activity was monitored over time. In (C) and (D), the 48-72 h time points were lost due to an error during sample preparation. All four independent biological replicates (A-D) were used to calculate the fold-change in Figure 1C. The limit of detection is indicated.

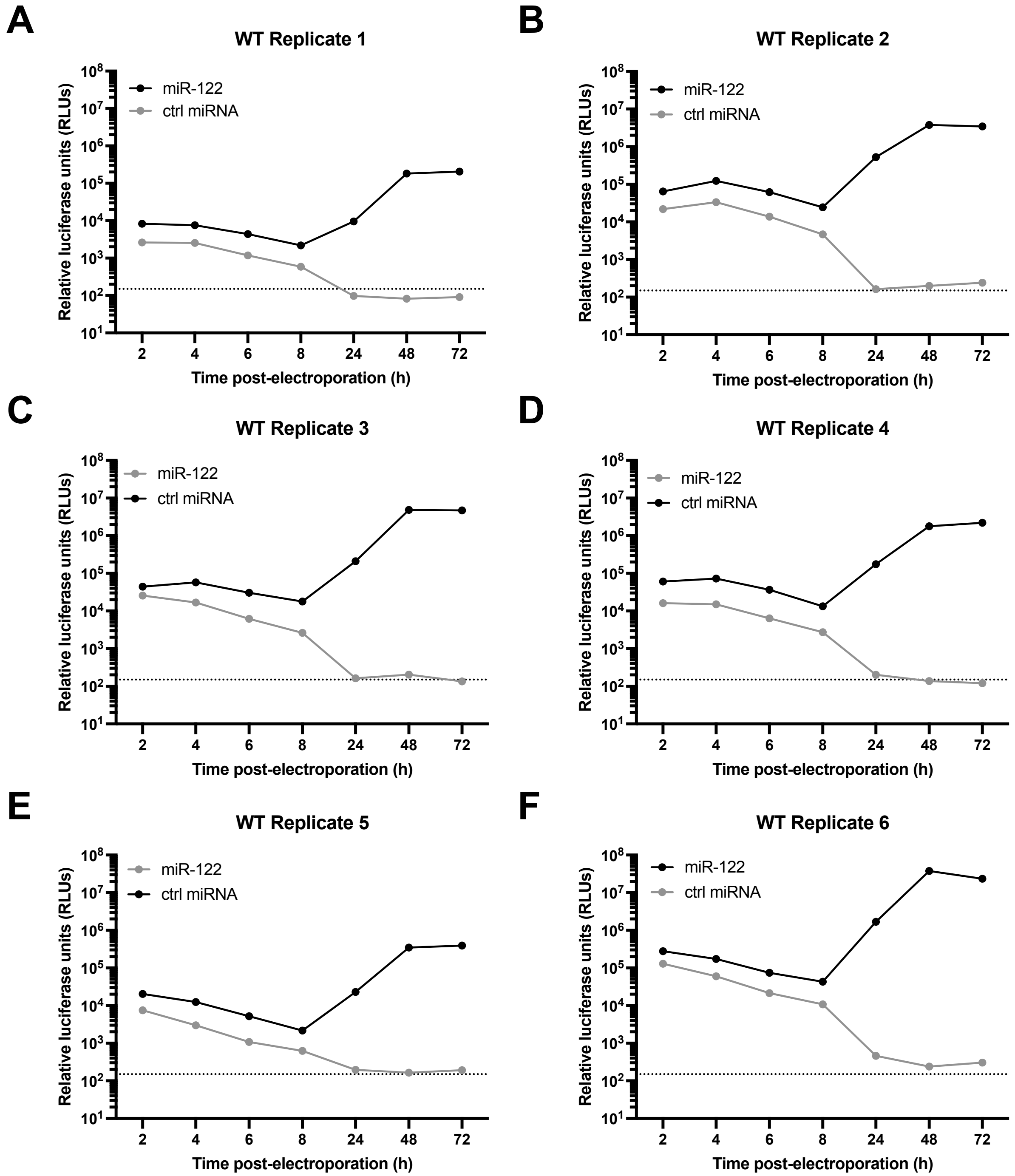

Figure S2. Independent biological replicate (WT) data for the overall contributions of miR-122's riboswitch, genome stability, and translation promotion activities. (A-F) Full-length RLuc HCV RNAs were co-electroporated into miR-122 knockout (KO) cells with miR-122 or control (ctrl) miRNA, and a capped Firefly luciferase (FLuc) reporter RNA. Half the number of cells were plated for the 24-72 h time points and the 48-72 h lysates were diluted 2-fold to ensure values were within range of the luciferase assay. RLuc activity was monitored over time. All six independent biological replicates (A-F) were used to calculate the fold-change in Figure 1F. The limit of detection is indicated.

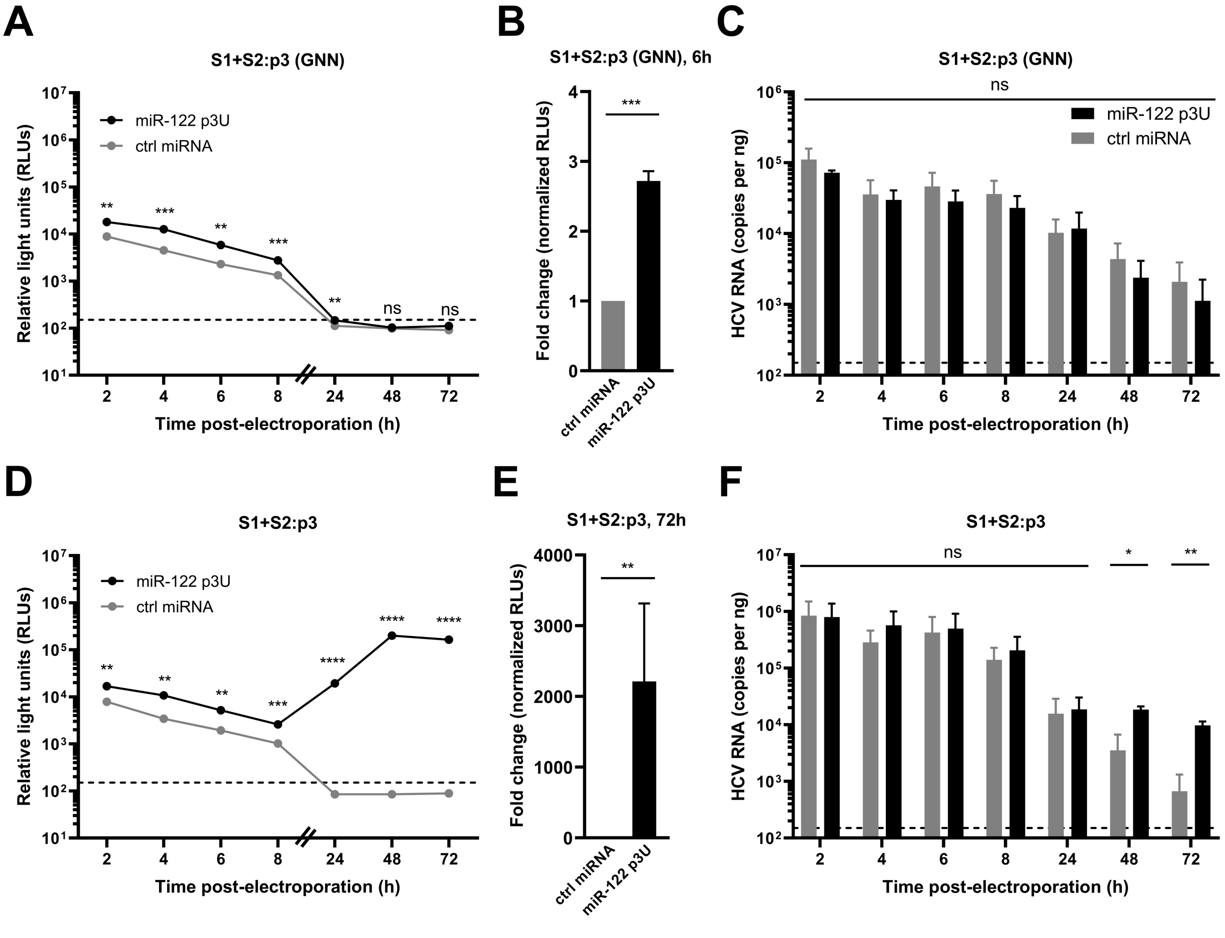

Figure S3. Alternative quantification of the overall contributions of miR-122's riboswitch, genome stability and translational promotion activities in Huh-7.5 cells. **(A-F)** Full-length RLuc HCV S1+S2:p3 RNAs were co-electroporated into Huh-7.5 cells with miR-122 p3U or control (ctrl) miRNA, and a capped Firefly luciferase (FLuc) reporter RNA. RLuc activities for **(A-B)** S1+S2:p3 (GNN) and **(D-E)** S1+S2:p3 viral RNAs were monitored over time. The limit of detection is indicated. Data in **(A)** and **(D)** is representative data from one of three independent biological replicates with three technical replicates, and error bars represent the standard error of the mean (SEM). The limit of detection is indicated. In **(B)** and **(E)**, RLuc activity for S1+S2:p3 (GNN) or S1+S2:p3 HCV RNAs at 6 h or 72 h, respectively, normalized to the FLuc (transfection efficiency) control at 2 h, were used to calculate the fold change, with the control miRNA condition set to 1. Data is displayed as the mean of four (S1+S2:p3 GNN) and three (S1+S2:p3) independent biological replicates, and error bars represent the SEM. Viral RNA levels for **(C)** S1+S2:p3 (GNN) and **(F)** S1+S2:p3 HCV were monitored by RT-qPCR (as described in *Figure 1*). The limit of detection is indicated. Data is displayed as the mean of three independent biological replicates (except for the 6h S1+S2:p3 (GNN) + ctrl miRNA and 72h S1+S2:p3 (GNN) + miR-122 p3U conditions which represent two independent replicates), with error bars corresponding to the SEM. Statistical significance was determined by multiple Student's *t* test, ****$p \leq 0.0001$; *** $p \leq 0.001$; **$p \leq 0.01$; *$p \leq 0.05$ ns, not significant ($p \geq 0.05$).

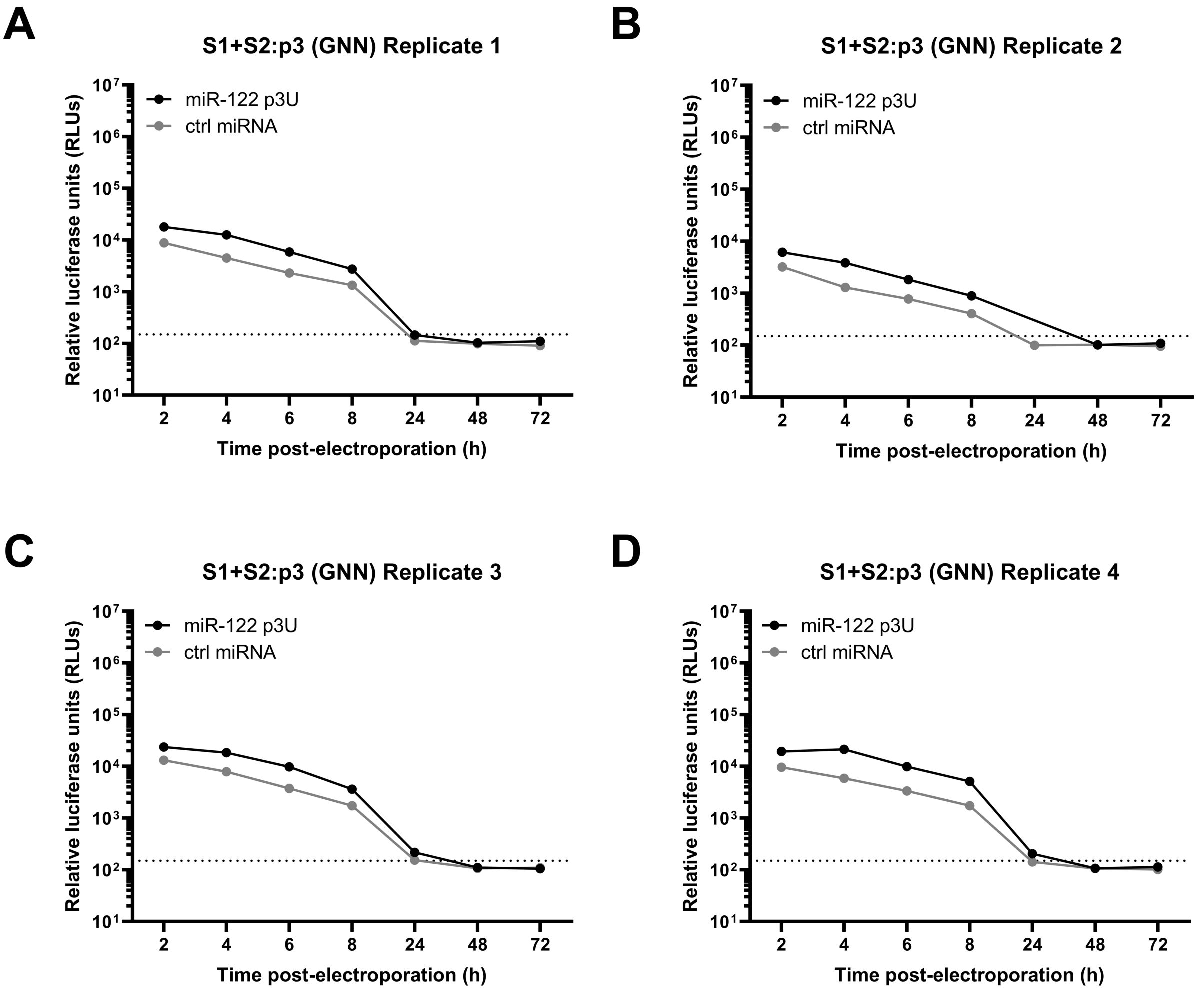

Figure S4. Independent biological replicate (S1+S2:p3 GNN) data for the alternative quantification of the overall contribution of miR-122's riboswitch, genome stability, and translation promotion activities in Huh-7.5 cells. (A-D) Full-length RLuc HCV S1+S2:p3 (GNN) RNAs were co-electroporated into Huh-7.5 cells with miR-122 p3U or control (ctrl) miRNA, and a capped Firefly luciferase (FLuc) reporter RNA. RLuc activity was monitored over time. In (B), the 24 h time point was lost due to an error during sample preparation. All four independent biological replicates (A-D) were used to calculate the fold-change in Figure S3B. The limit of detection is indicated.

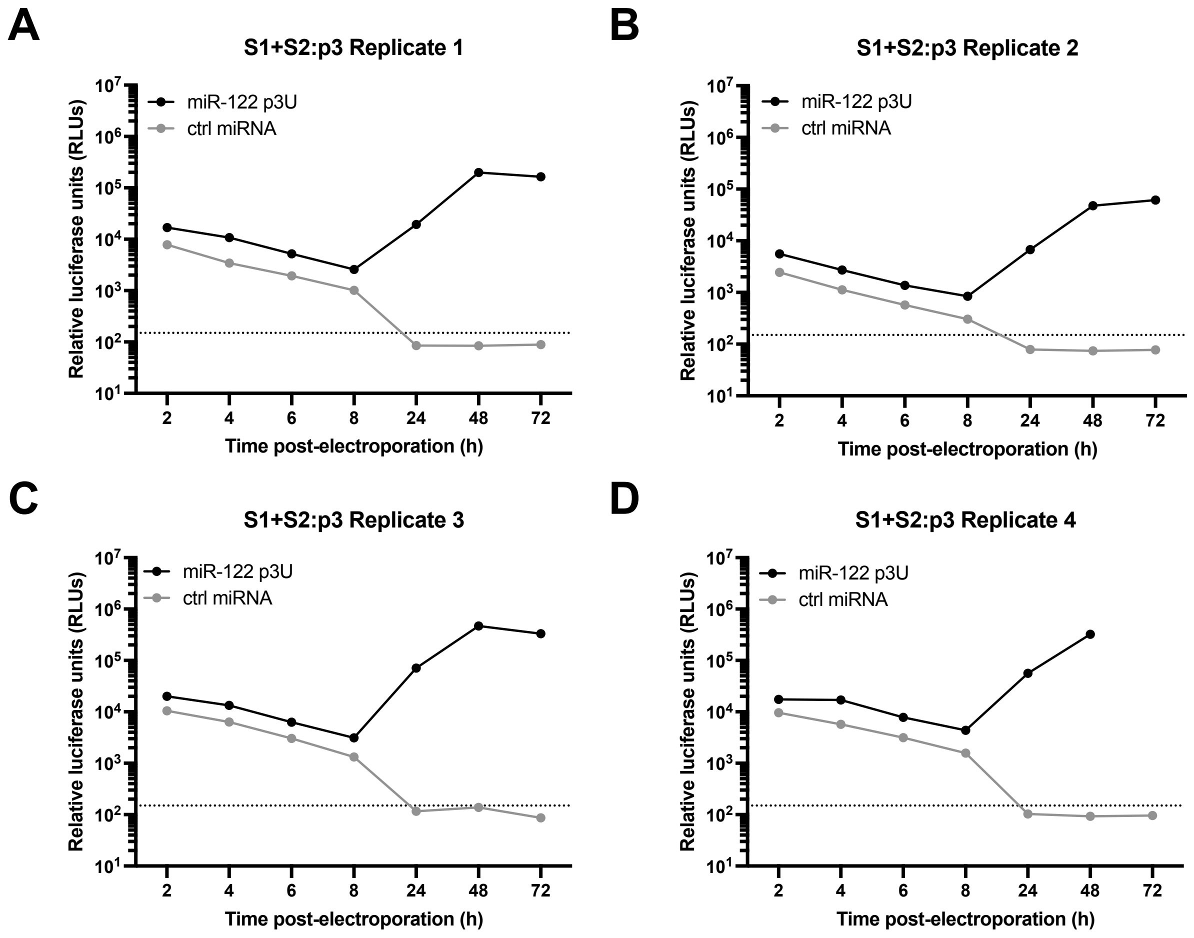

Figure S5. Independent biological replicate (S1+S2:p3) data for the alternative quantification of the overall contribution of miR-122's riboswitch, genome stability, and translation promotion activities in Huh-7.5 cells. (A-D) Full-length RLuc HCV S1+S2:p3 RNAs were co-electroporated into Huh-7.5 cells with miR-122 p3U or control (ctrl) miRNA, and a capped Firefly luciferase (FLuc) reporter RNA. Half the number of cells were plated for the 24-72 h time points and the 48-72 h lysates were diluted 2-fold to ensure values were within range of the luciferase assay. RLuc activity was monitored over time. In (D), the 72 h time point was lost due to an error during sample preparation. As such, three independent biological replicates (A-C) were used to calculate the fold-change in Figure S3E. The limit of detection is indicated.

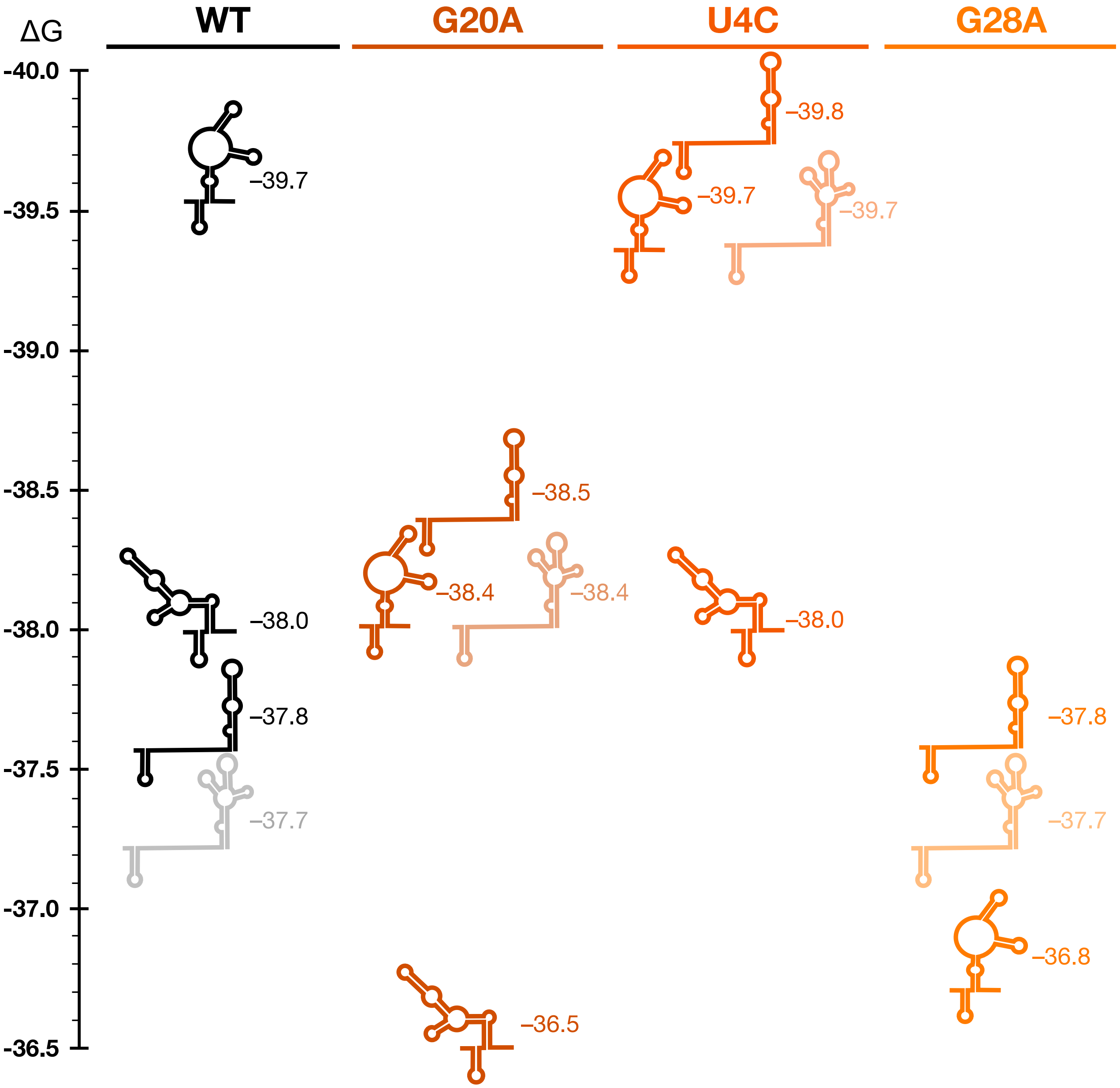

Figure S6. RNA structure predictions for the first 117 nucleotides of the WT, G20A, U4C and G28A JFH-1 genomes. Predicted RNA secondary structures and their associated Gibb's free energy (ΔG) predictions were calculated using RNAStructure, with the first 3 nucleotides constrained as single-stranded.

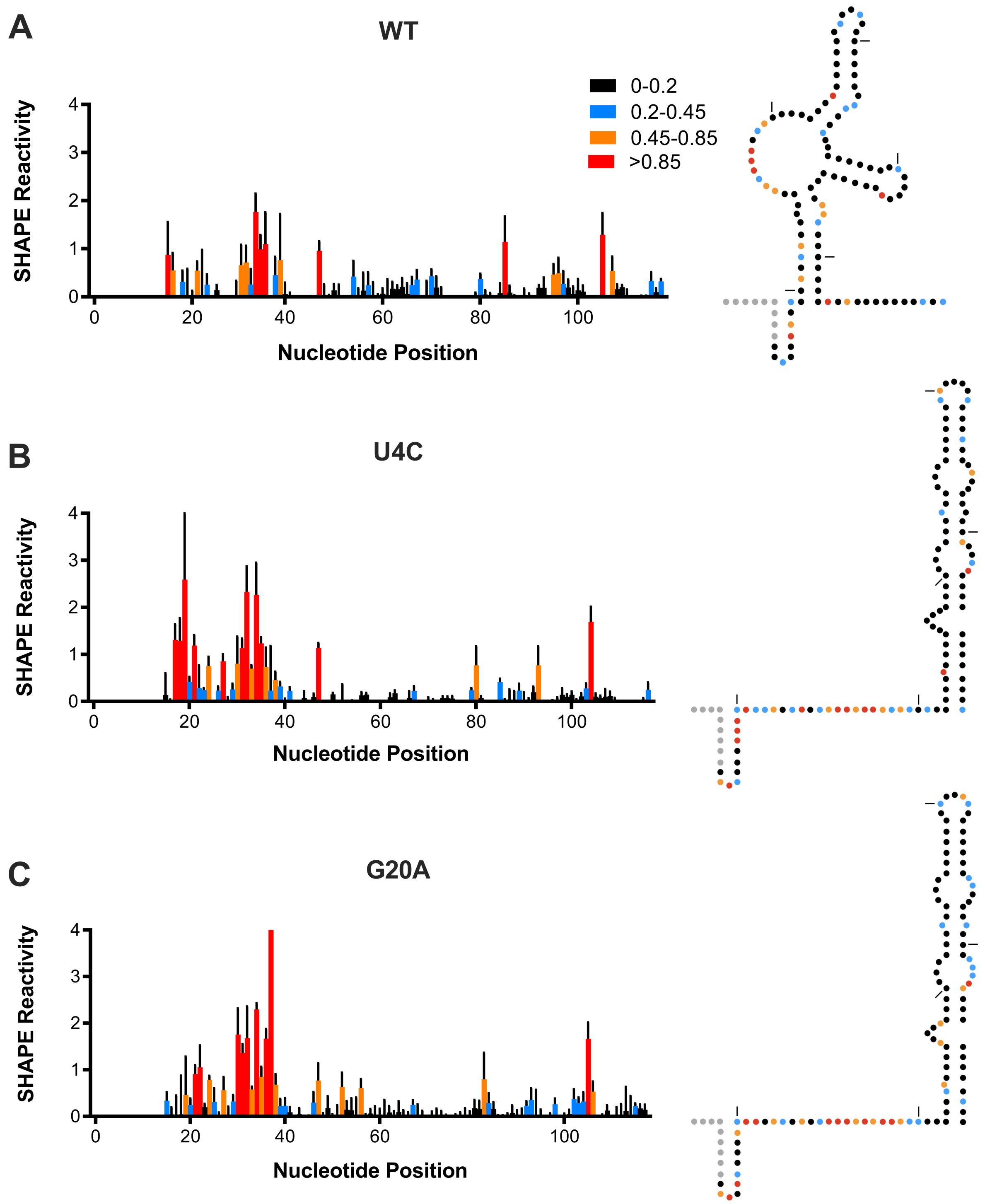

Figure S7. ***In vitro*** **SHAPE analysis of the 5′ terminus of WT, U4C and G20A HCV RNAs.** Normalized SHAPE reactivities of nucleotides 1-117 of **(A)** WT, **(B)** U4C, and **(C)** G20A HCV RNAs (*Left*). Nucleotides with high (≥ 0.85, red), intermediate (0.45 - 0.85, orange), low (0.2 - 0.45, blue) and very low (≤ 0.2) SHAPE reactivity are indicated. Nucleotides 1-9 were omitted due to high background reactivity. Data displayed is mean SHAPE reactivity representative of four biological replicates and error bars represent standard error of the mean. Prediction of the lowest free energy structure formed by the first 117 nt of the HCV genome for each variant constrained by SHAPE reactivity is depicted via dot plot (*Right)* with relative SHAPE reactivity superimposed. Tick marks represent 20 nucleotide intervals.

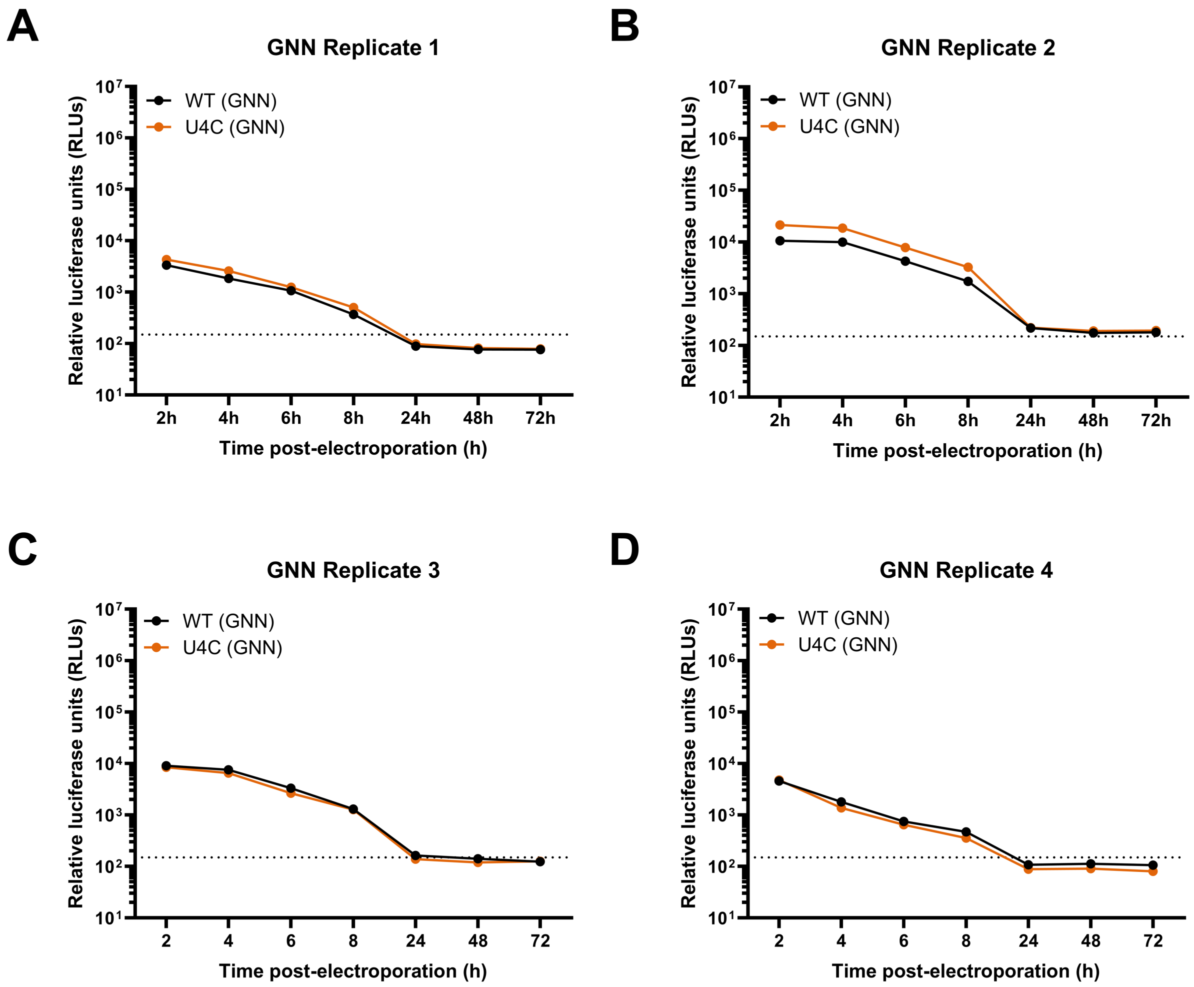

Figure S8. Independent biological replicate data for the riboswitch activity using GNN vs. U4C GNN. (A-D) Full-length RLuc HCV (GNN) and U4C (GNN) RNAs were co-electroporated into miR-122 KO cells and a capped Firefly luciferase (FLuc) reporter RNA. RLuc activity was monitored over time. All four independent biological replicates (A-D) were used to calculate the fold-change in Figure 2C. The limit of detection is indicated.

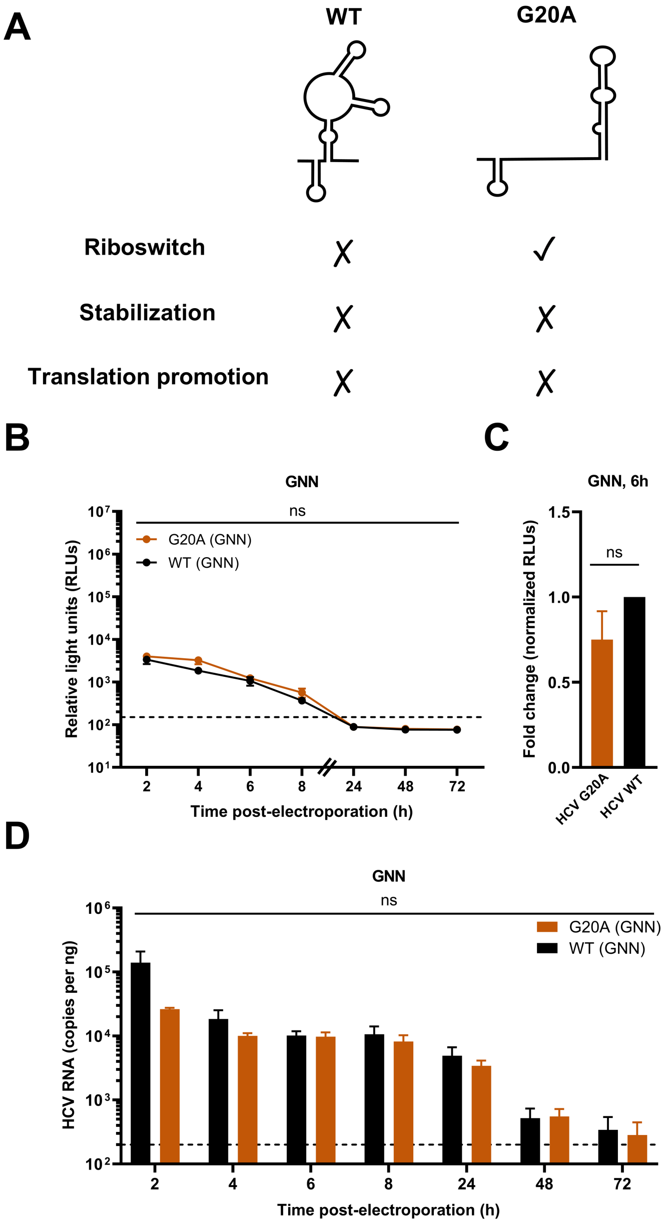

Figure S9. Alternative quantification of the riboswitch effect using the G20A mutant. (A) Schematic representation of the experimental set-up for the riboswitch activity assay. In the absence of miR-122, WT HCV RNA favors the SLII^{alt} conformation, while the G20A mutant favors the "riboswitched" (SLII) conformation. In the absence of miR-122, the stabilization and translational promotion roles of miR-122 are not fulfilled in either condition, allowing for the isolation of the riboswitch effect. **(B)** Full-length RLuc WT (GNN) or G20A (GNN) HCV RNAs were co-electroporated into miR-122 KO cells with a capped FLuc reporter RNA. RLuc activity was monitored over time. The data in **(B)** is representative data from one of four independent biological replicates with three technical replicates, and error bars represent the standard error of the mean (SEM). The limit of detection is indicated. **(C)** RLuc activity at 6 h normalized to Fluc (transfection efficiency) control at 2 h, was used to calculate the fold change between WT (GNN) and G20A (GNN), with the WT (GNN) condition set to 1. Data is displayed as the mean of four independent biological replicates, and error bars represent the SEM. **(D)** Viral RNA levels were monitored by RT-qPCR as described in *Figure 1*. The limit of detection is indicated. Data is displayed as the mean of three independent biological replicates, with error bars corresponding to the SEM. Statistical significance was determined by multiple Student's *t* test. ns, not significant ($p \geq 0.05$).

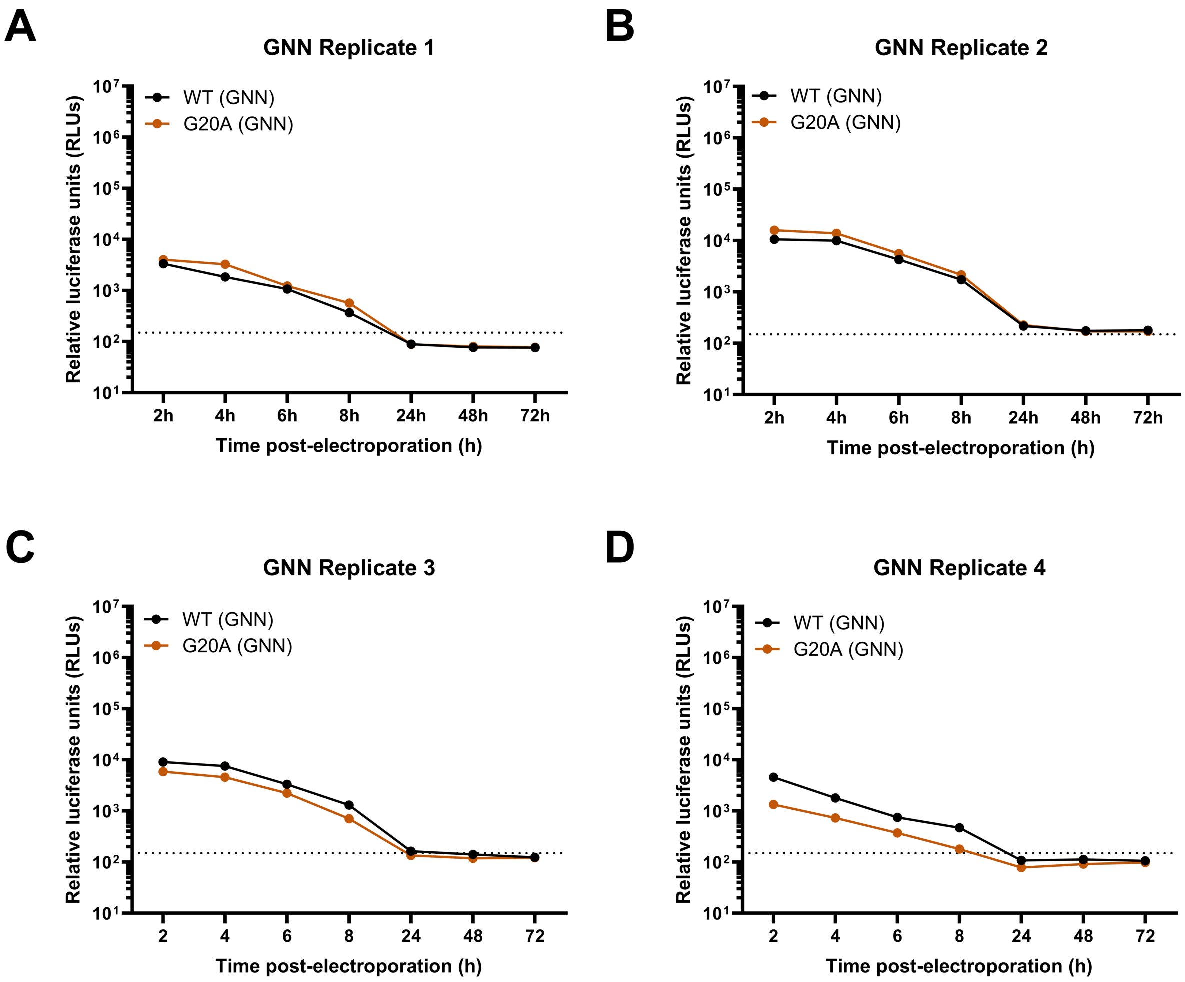

Figure S10. Independent biological replicate data for the riboswitch activity using GNN vs. G20A GNN. (A-D) Full-length RLuc HCV (GNN) and G20A (GNN) RNAs were co-electroporated into miR-122 KO cells and a capped Firefly luciferase (FLuc) reporter RNA. RLuc activity was monitored over time. All four independent biological replicates (A-D) were used to calculate the fold-change in Figure S9C. The limit of detection is indicated.

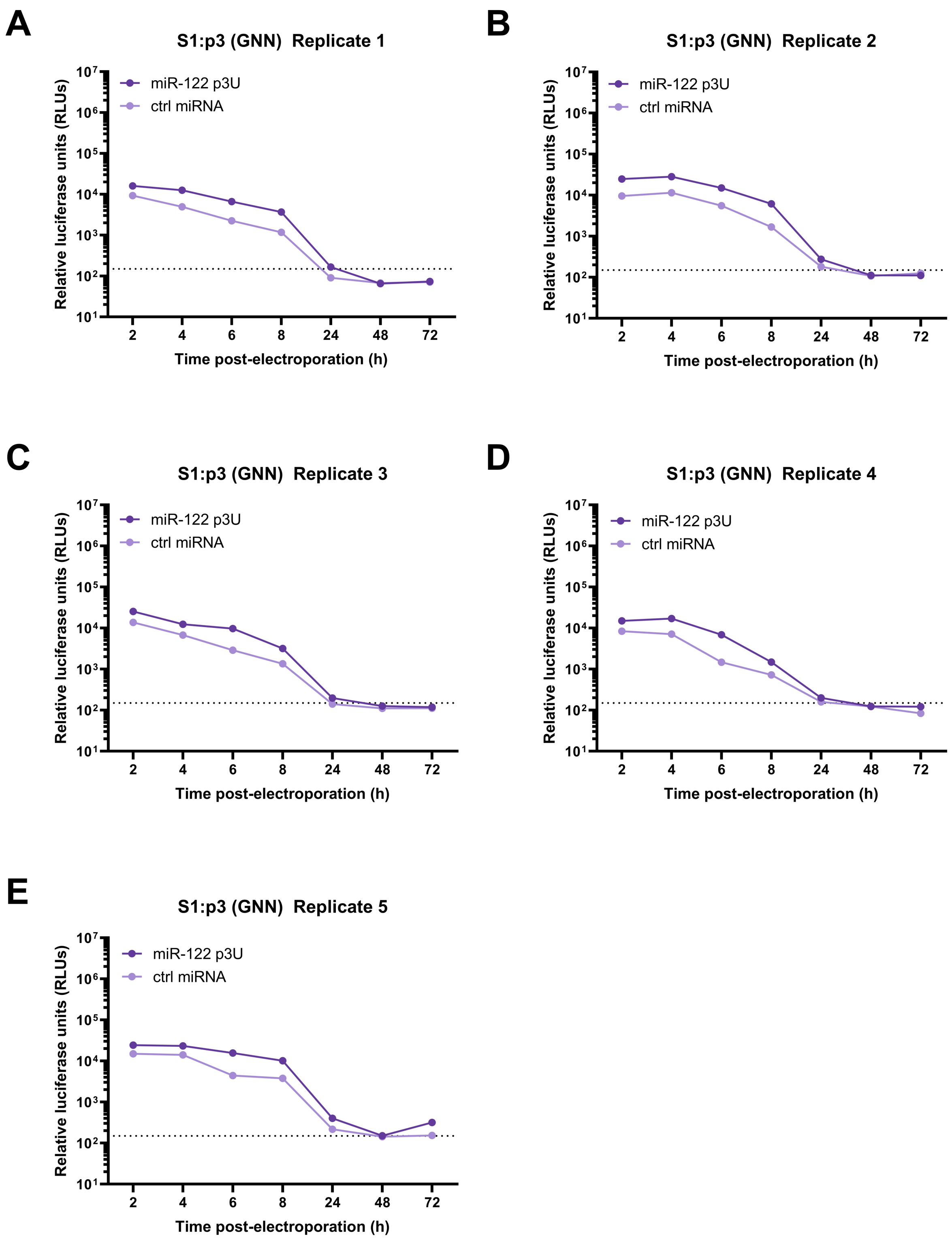

Figure S11. Independent biological replicate (S1:p3 GNN) data for genome stability experiments. (A-E) Full-length RLuc S1:p3 (GNN) HCV RNAs were co-electroporated into Huh-7.5 cells with miR-122 p3U or control (ctrl) miRNA, and a capped Firefly luciferase (FLuc) reporter RNA. RLuc activity was monitored over time. All five independent biological replicates (A-E) were used to calculate the fold-change in Figure 3C. The limit of detection is indicated.

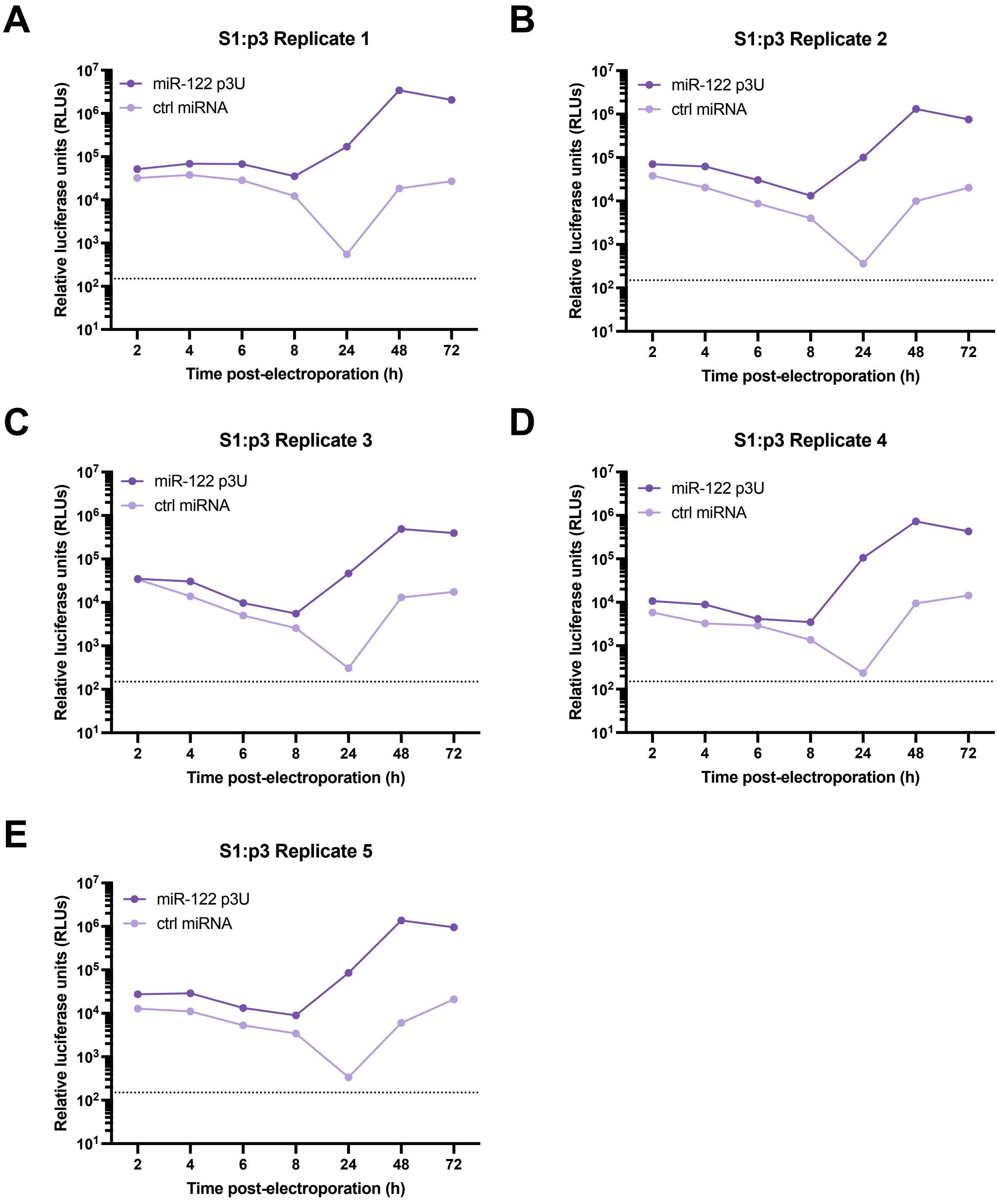

Figure S12. Independent biological replicate (S1:p3) data for genome stability experiments. (A-E) Full-length RLuc S1:p3 HCV RNAs were co-electroporated into Huh-7.5 cells with miR-122 p3U or control (ctrl) miRNA, and a capped Firefly luciferase (FLuc) reporter RNA. RLuc activity was monitored over time. All five independent biological replicates (A-E) were used to calculate the fold-change in Figure 3F. The limit of detection is indicated.

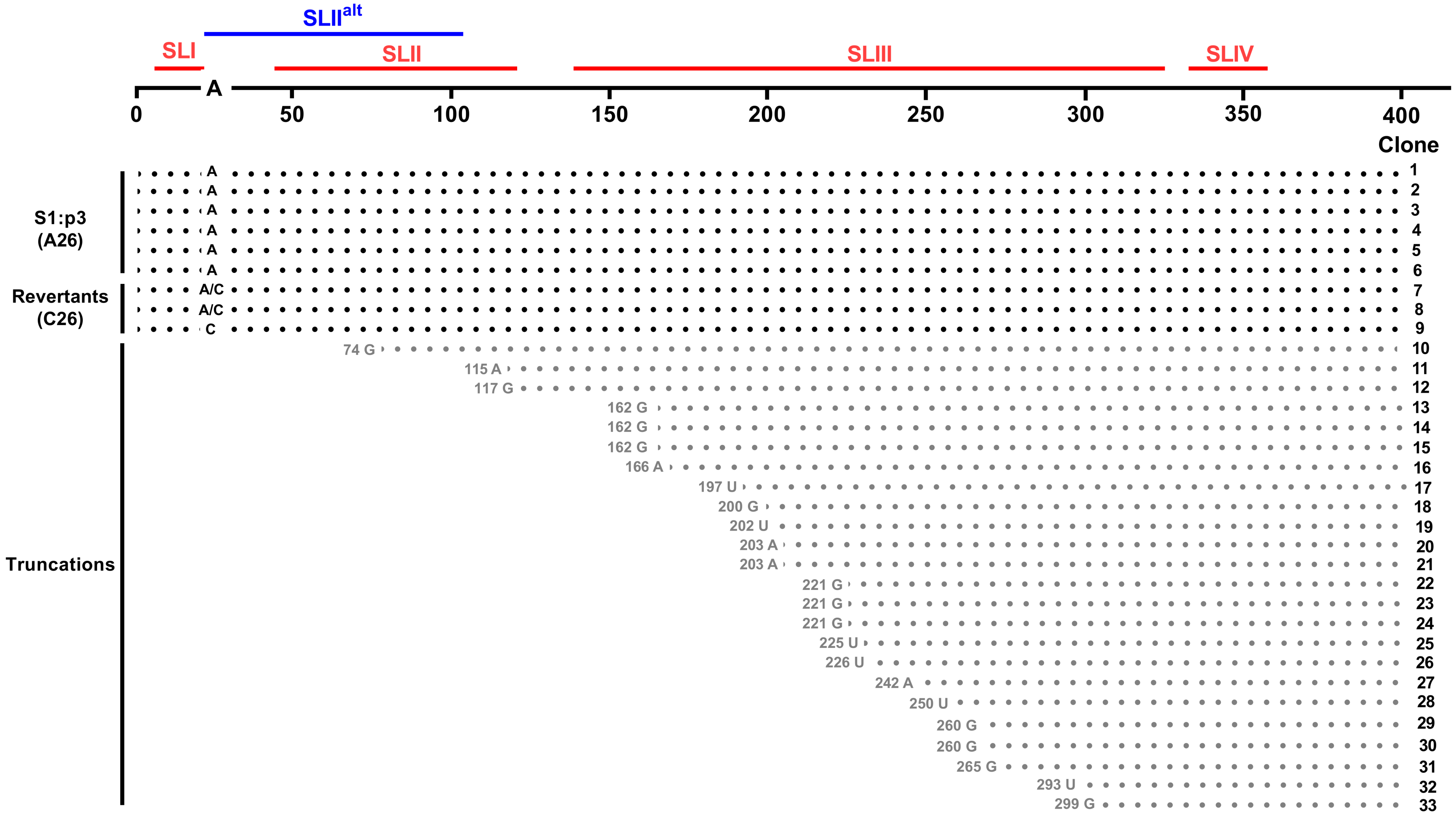

Figure S13. 5′ Rapid amplification of cDNA ends (RACE) to confirm the identity of the HCV 5′ terminus. 5′ RACE was performed on RNA obtained at the 72 h end point of the stability assay (Figure 3) and a total of 33 clones were sequenced. Each sequence obtained is represented by a dotted line, with the nucleotide identity at position 26 indicated when applicable, and the 5′ terminal nucleotide identity and position indicated for truncated products. No other mutations were identified in the region of the genome sequenced.

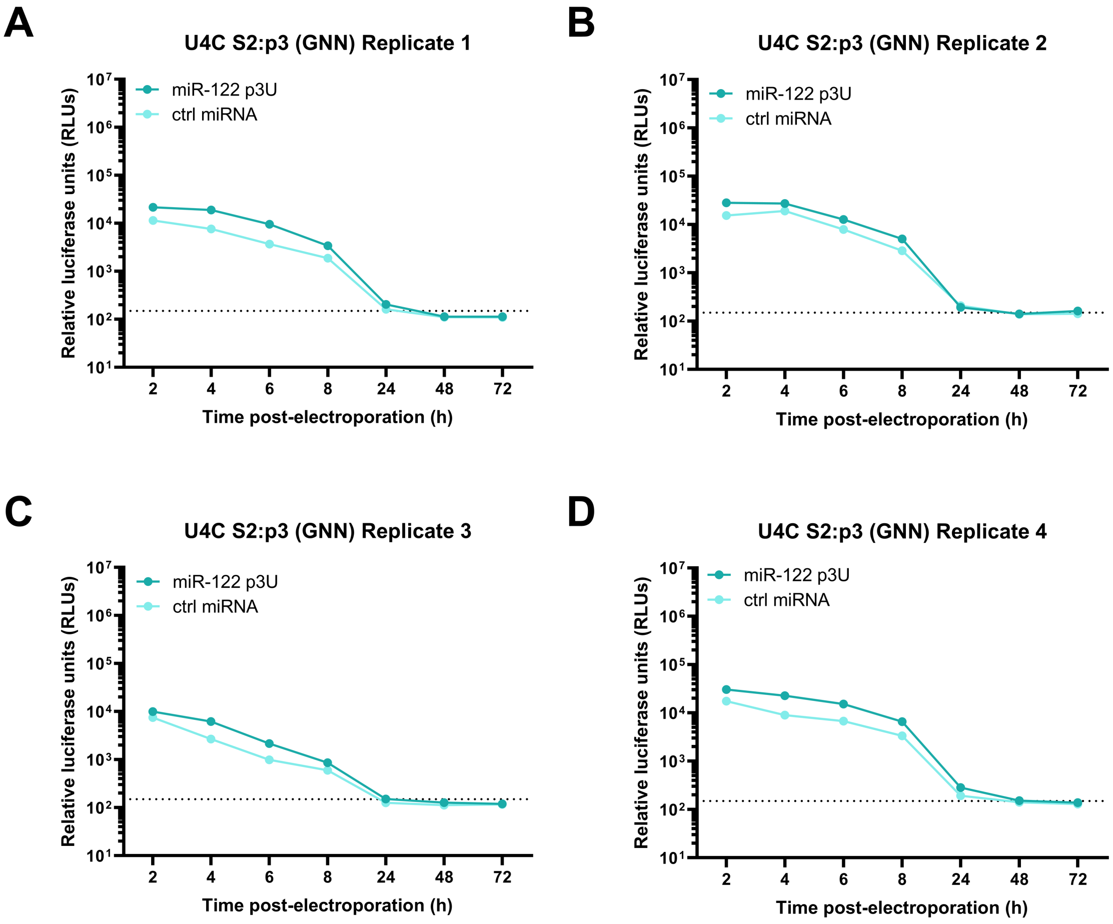

Figure S14. Independent biological replicate (U4C S2:p3 GNN) data for translational promotion experiments. (A-D) Full-length RLuc U4C S2:p3 (GNN) HCV RNAs were co-electroporated into Huh-7.5 cells with miR-122 p3U or control (ctrl) miRNA, and a capped Firefly luciferase (FLuc) reporter RNA. RLuc activity was monitored over time. All four independent biological replicates (A-D) were used to calculate the fold-change in Figure 4C. The limit of detection is indicated.

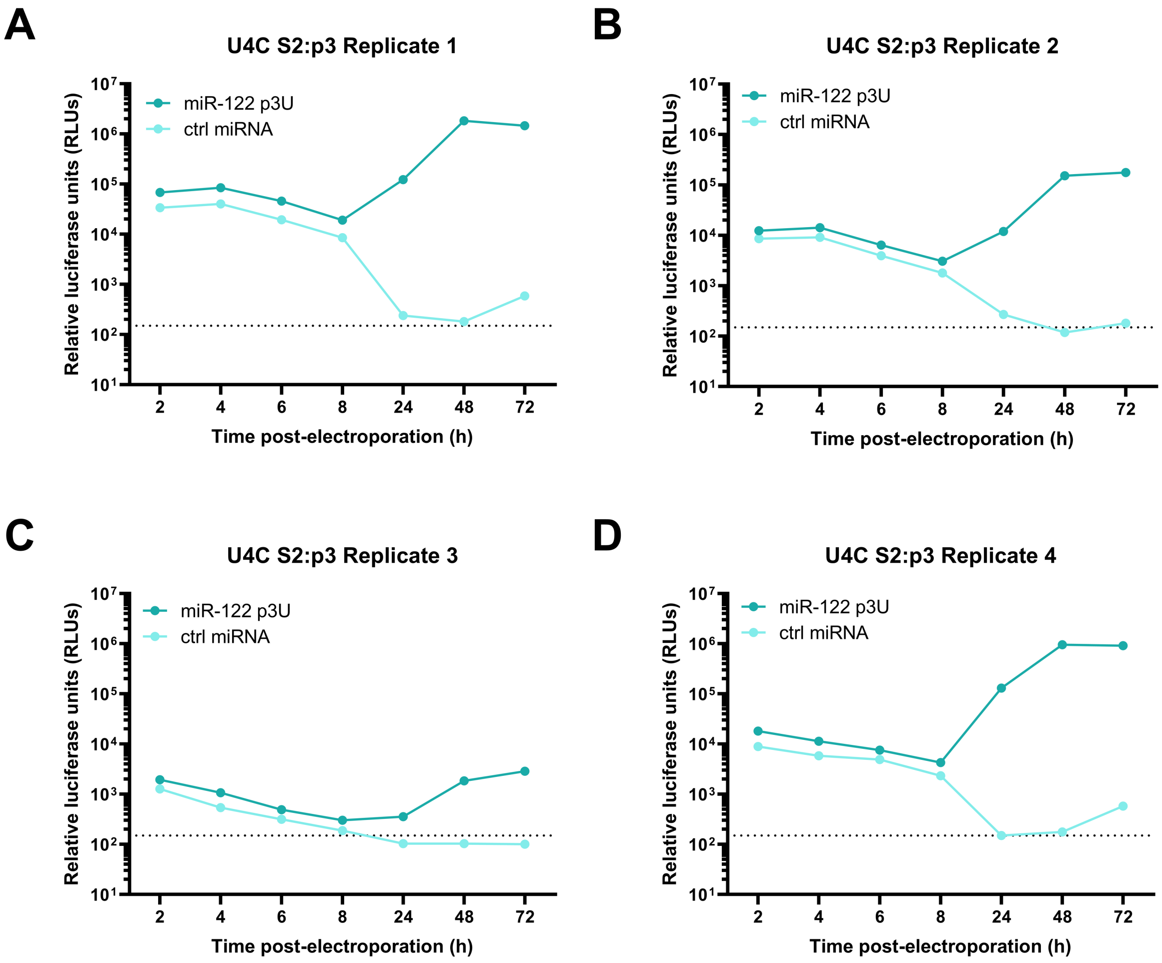

Figure S15. Independent biological replicate (U4C S2:p3) data for translational promotion experiments. (A-D) Full-length RLuc U4C S2:p3 HCV RNAs were co-electroporated into Huh-7.5 cells with miR-122 p3U or control (ctrl) miRNA, and a capped Firefly luciferase (FLuc) reporter RNA. RLuc activity was monitored over time. All four independent biological replicates (A-D) were used to calculate the fold-change in Figure 4C. The limit of detection is indicated.

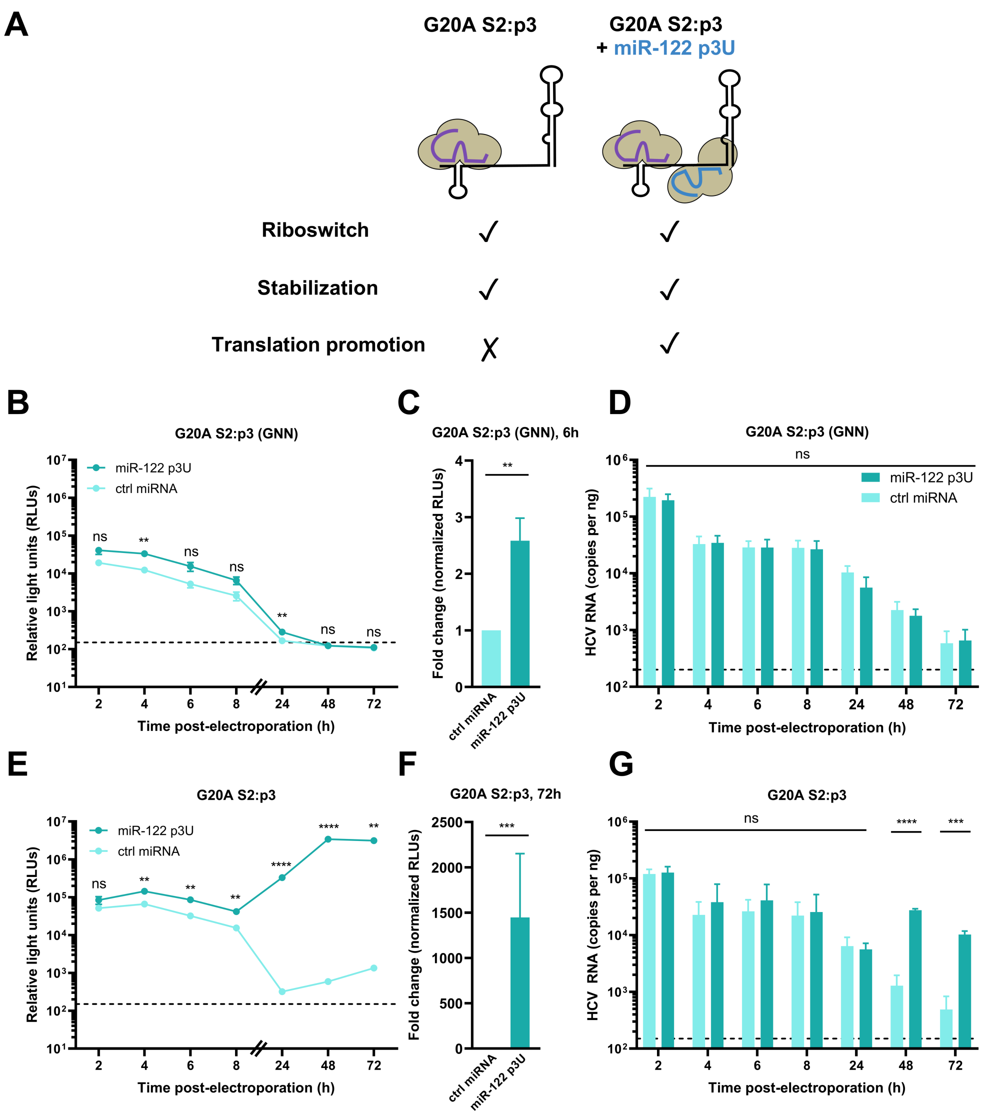

Figure S16. Alternative quantification of the translational promotion effect using G20A S2:p3 mutant. (A) Schematic representation of the experimental set-up for translational promotion assays. HCV G20A S2:p3 is riboswitched *a priori*, and endogenous miR-122 can bind to site 1, thereby fulfilling the stabilization effect. Addition of miR-122 p3U allows binding to site 2 and measurement of the translational promotion effect in isolation. **(B-G)** Full-length RLuc HCV G20A S2:p3 RNAs were co-electroporated into Huh-7.5 cells with miR-122 p3U or ctrl miRNA, as well as a capped FLuc reporter RNA. RLuc activities for **(B-C)** G20A S2:p3 (GNN) or **(E-F)** G20A S2:p3 viral RNAs were monitored over time (as described in *Figure 1*). The limit of detection is indicated. Data in **(B)** and **(E)**, are representative data from one of four independent biological replicates with three technical replicates, and error bars represent the standard error of the mean (SEM). In **(C)** and **(F)**, the RLuc activity for G20A S2:p3 (GNN) or G20A S2:p3 HCV RNAs at 6 h or 72 h, respectively, normalized to FLuc (transfection efficiency) control at 2 h, were used to calculate the fold change, with the control miRNA condition set to 1. The limit of detection is indicated. Data displayed is the mean of four independent biological replicates, and error bars represent the SEM. Viral RNA levels for **(D)** G20A S2:p3 (GNN) and **(G)** G20A S2:p3 HCV were monitored by RT-qPCR (as in described in *Figure 1*). The limit of detection is indicated. Data is displayed as the mean of three independent biological replicates with error bars corresponding to the SEM. Statistical significance was determined by multiple Student's *t* test, ****$p \leq 0.0001$; *** $p \leq 0.001$; **$p \leq 0.01$; *$p \leq 0.05$ ns, not significant ($p \geq 0.05$).

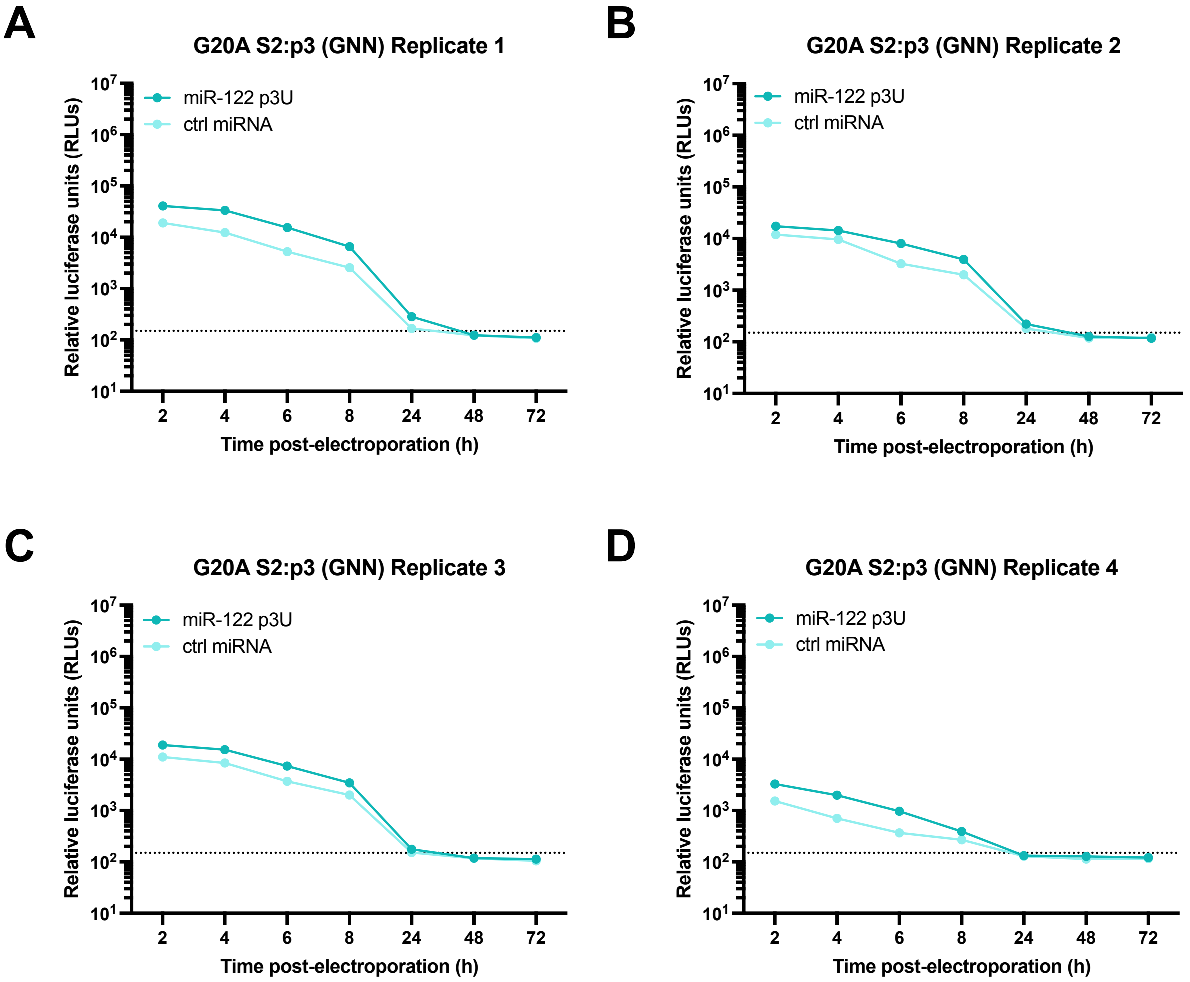

Figure S17. Independent biological replicate (G20A S2:p3 GNN) data for translational promotion experiments. (A-D) Full-length RLuc G20A S2:p3 GNN HCV RNAs were co-electroporated into Huh-7.5 cells with miR-122 p3U or control (ctrl) miRNA, and a capped Firefly luciferase (FLuc) reporter RNA. RLuc activity was monitored over time. All four independent biological replicates (A-D) were used to calculate the fold-change in Figure S16C. The limit of detection is indicated.

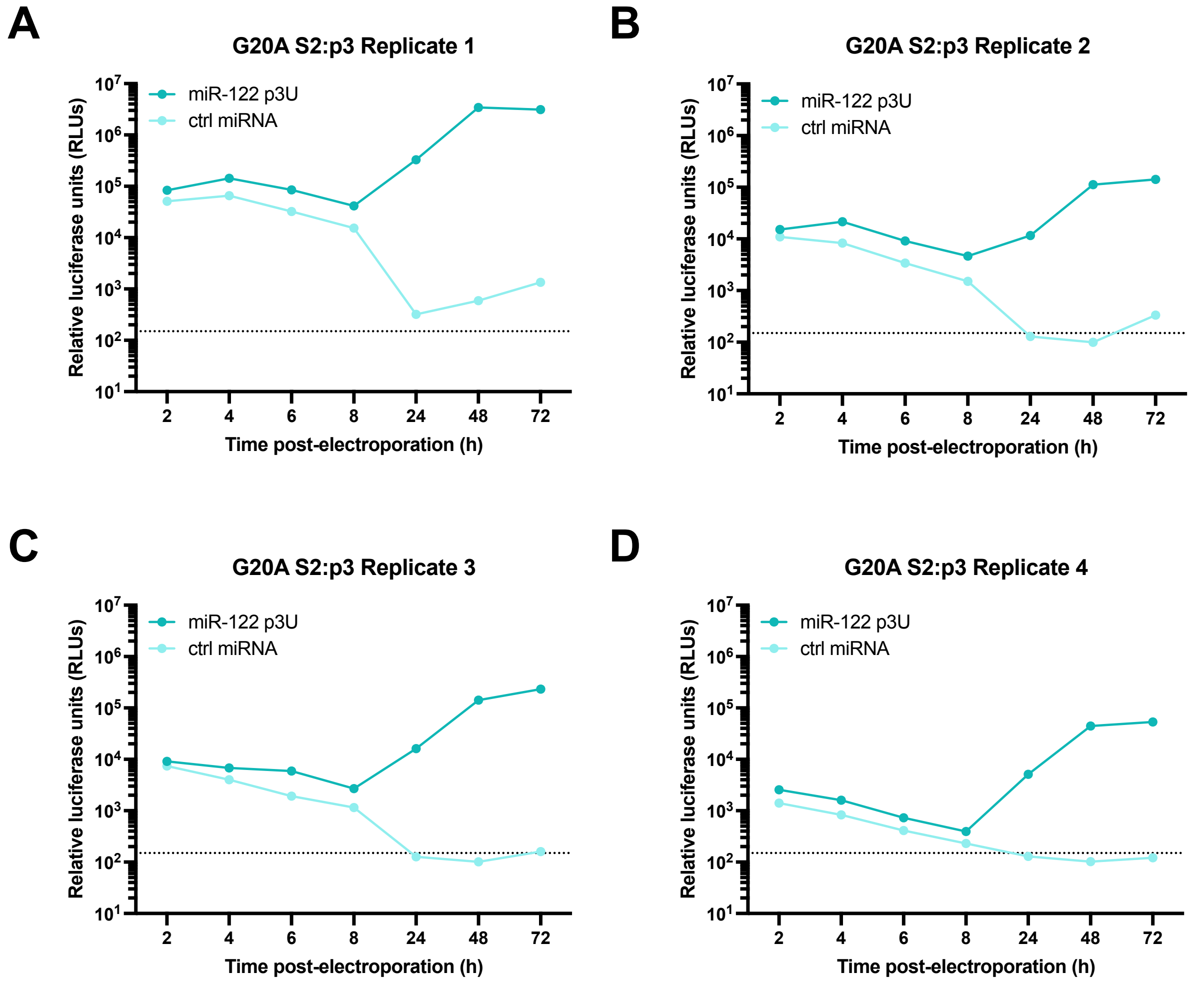

Figure S18. Independent biological replicate (G20A S2:p3) data for translational promotion experiments. (A-D) Full-length RLuc G20A S2:p3 HCV RNAs were co-electroporated into Huh-7.5 cells with miR-122 p3U or control (ctrl) miRNA, and a capped Firefly luciferase (FLuc) reporter RNA. RLuc activity was monitored over time. All four independent biological replicates (A-D) were used to calculate the fold-change in Figure S16F. The limit of detection is indicated.

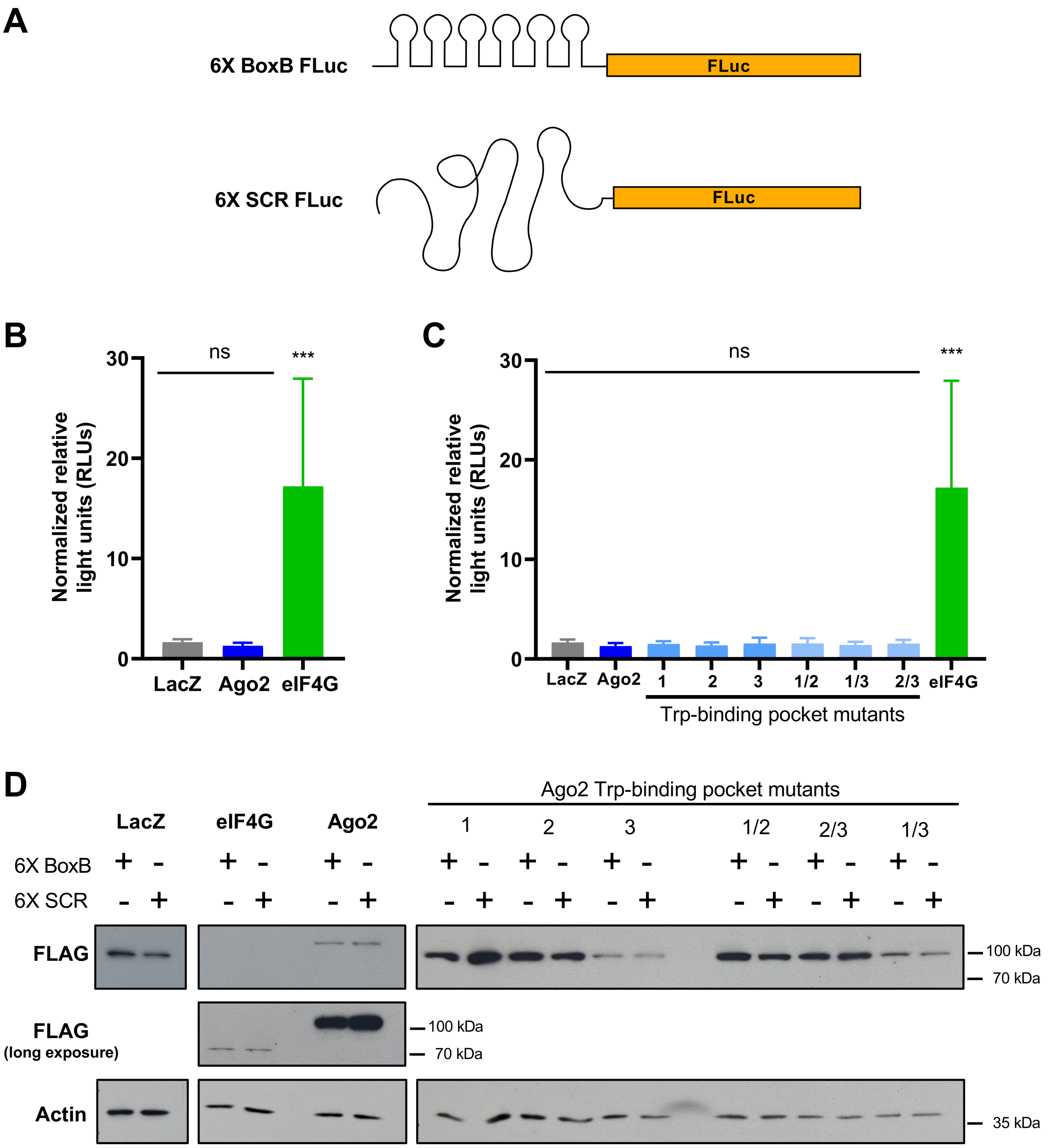

Figure S19. Tethering Ago2 to the 5′ UTR of a reporter RNA does not promote translation. (A) Schematic representation of 6X BoxB and 6X scrambled (SCR) reporter mRNAs. **(B-D)** Individual λN-fusion protein encoding plasmids were co-transfected in HEK 293T cells with a control RLuc reporter encoding plasmid. One day post-transfection, cells were transfected with 6X BoxB or 6X scr FLuc reporter RNAs and harvested for Western blot and luciferase assay at 16 h post-transfection. **(B)** Luciferase activity of WT λN-Ago2, λN-LacZ and λN-eIF4G conditions. **(C)** Luciferase activity for the WT and mutant λN-Ago2 constructs. Luciferase activity is presented as FLuc normalized to the RLuc control, and all conditions were further normalized to the LacZ scr control condition. Normalized luciferase signal is reported as the mean of three independent biological replicates, and error bars represent to the SD of the mean. Statistical significance was determined by multiple Student's *t* test, $***p \leq 0.0001$; ns, not significant ($p \geq 0.01$). **(D)** Expression of WT and mutant λN-Ago2, λN-LacZ and λN-eIF4G was verified by Western blot.
